# TGFBI modulates tumour hypoxia and promotes breast cancer metastasis

**DOI:** 10.1002/1878-0261.12828

**Published:** 2020-11-05

**Authors:** Flavia Fico, Albert Santamaria‐Martínez

**Affiliations:** ^1^ Tumor Ecology Lab Department of Oncology, Microbiology and Immunology Faculty of Science and Medicine University of Fribourg Switzerland

**Keywords:** breast cancer, cancer stem cells, hypoxia, metastasis, TGFBI

## Abstract

Breast cancer metastasis is a complex process that depends not only on intrinsic characteristics of metastatic stem cells, but also on the particular microenvironment that supports their growth and modulates the plasticity of the system. In search for microenvironmental factors supporting cancer stem cell (CSC) growth and tumour progression to metastasis, we here investigated the role of the matricellular protein transforming growth factor beta induced (TGFBI) in breast cancer. We crossed the MMTV‐PyMT model of mammary gland tumorigenesis with a *Tgfbi* ^Δ/Δ^ mouse and studied the CSC content of the tumours. We performed RNAseq on wt and ko tumours, and analysed the tumour vasculature and the immune compartment by IHC and FACS. The source of TGFBI expression was determined by qPCR and by bone marrow transplantation experiments. Finally, we performed *in silico* analyses using the METABRIC cohort to assess the potential prognostic value of TGFBI. We observed that deletion of *Tgfbi* led to a dramatic decrease in CSC content and lung metastasis. Our results show that lack of TGFBI resulted in tumour vessel normalisation, with improved vessel perfusion and decreased hypoxia, a major factor controlling CSCs and metastasis. Furthermore, human data mining in a cohort of breast cancer patients showed that higher expression of *TGFBI* correlates with poor prognosis and is associated with the more aggressive subtypes of breast cancer. Overall, these data reveal a novel biological mechanism controlling metastasis that could potentially be exploited to improve the efficacy and delivery of chemotherapeutic agents in breast cancer.

## Graphical abstract

The ECM is an essential component of the tumour microenvironment and plays important functions during metastatic colonisation. Depletion of the matricellular protein TGFBI in MMTV‐PyMT mice normalises the vasculature and reduces hypoxia. As a result, *Tgfbi* loss leads to a dramatic decrease in CSC content and lung metastasis, unveiling its potential as a novel target in breast cancer.

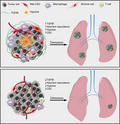

**Abbreviations**
CSC: cancer stem cell
ECM: extracellular matrix
EMT: epithelial‐to‐mesenchymal transition
FACS: fluorescence‐activated cell sorting
TGFBI: transforming growth factor beta induced

## 1 Introduction

Many cancers are organised as a hierarchy in which the so‐called cancer stem cells (CSC) can give rise to both CSCs and a differentiated progeny [1]. CSCs sustain tumour growth, and subsets of CSCs are responsible for metastatic colonisation [2, 3, 4, 5, 6], which is especially relevant since in most solid tumours, metastasis represents the late steps of tumour progression and is the main cause of death by cancer. The degree of plasticity of the CSC pool is a reflection of both intrinsic cellular properties and external signals derived from the tumour microenvironment [1]. Indeed, we and others have shown that specific components of the tumour microenvironment, in particular extracellular matrix (ECM) proteins, have essential functions during metastatic colonisation [4, 7, 8].

The ECM is a highly dynamic and complex network of biochemically discrete elements such as proteins, glycoproteins, polysaccharides and proteoglycans. Besides its fundamental role in maintaining tissue morphology, the ECM is known to control mechanotransduction, to modulate tumour angiogenesis and to provide a suitable niche for CSCs [9, 10]. Due to its glycosylated and charged nature, the ECM can bind many secreted growth factors and molecules [11], thus regulating their distribution and availability and therefore influencing signalling activity. The role of the ECM in promoting tumour angiogenesis is well established, with many ECM fragments having angiostatic or angiogenic properties [12]. Importantly, aberrant tumour angiogenesis is typically associated with hypoxia, a process that has been linked to the activation of a number of signalling pathways that govern CSC maintenance and expansion [13, 14]. The above‐mentioned ability of the ECM to generate localised sources of certain molecules or growth factors, its composition, its topography and its role in tumour angiogenesis are all factors that will determine the properties of the CSC niche and therefore the potential of a given tumour to colonise secondary organs.

Here, we aimed at clarifying the niche‐supporting role of the ECM protein transforming growth factor beta induced (TGFBI) in breast cancer. TGFBI is a conserved, fasciclin family, matricellular protein that has important functions during development, including in tissue branching morphogenesis and mesoderm differentiation in vertebrates, and somitogenesis in zebrafish [15, 16, 17, 18]. Our findings reveal that TGFBI plays an important role in tumour angiogenesis, thereby affecting tumour hypoxia and immune cell infiltration, which then ultimately generates a permissive microenvironment for CSCs and metastasis.

## 2 Methods

### 2.1 Antibodies and reagents

CD90.1 (HIS51), CD24 (M1/69), eBioscience (San Diego, CA, USA); TruStain FcX (CD16/32, clone 93), Ter119, CD8a (53‐6.7), CD11b (M1/70), CD31 (MEC13/3), CD44 (IM7), CD45 (30‐F11), F4/80 (BM8), BioLegend (San Diego, CA, USA); CD11b, Abcam (Cambridge, UK); mouse TGFBI, Merck‐Millipore (Burlington, MA, USA); human TGFBI, Thermo Scientific (Waltham, MA, USA); VINCULIN, Santa Cruz Biotechnology (Dallas, TX, USA); α‐SMA (1A4), CD31 (SP164), Sigma‐Aldrich (St. Louis, MO, USA); VIMENTIN, Lifespan Biosciences (Seattle, WA, USA); AldeFluor Assay, STEMCELL Technologies (Vancouver, Canada). All plasmids were produced by classical molecular cloning, and the *Tgfbi* sgRNAs were cloned into the lentiCRISPRv2 lentiviral vector as described previously [19]. The lentiCRISPRv2 was a gift from Feng Zhang (Addgene plasmid #52961). The sgRNA and Cas9 nuclease containing lentiviruses were used to infect C(3)TAg cells at low MOI and select them with 1 μg·mL^−1^ puromycin.

### 2.2 Cell culture

Mouse tumour tissue was dissociated mechanically, followed by an incubation with 1 : 66 Liberase TH (Roche, Basel, Switzerland) and DNAse (10 mg·mL^−1^) at 37 °C for 1 h. Cells were then washed twice in 2 mm EDTA in PBS and once in PBS and then plated in collagen‐coated plates (HBSS, BSA 100 mg·mL^−1^, HEPES 1 m pH 6.5 and bovine collagen biomatrix by Cell Systems). Cells were grown in DMEM:F12 (PAN Biotech, Aidenbach, Germany) supplemented with 2% FBS, 1% penicillin/streptomycin 20 ng·mL^−1^ EGF (Invitrogen, Carlsbad, CA, USA) and 10 μg·mL^−1^ insulin (Invitrogen) and let attach overnight. Human cell lines and 4T1 cells were obtained from the ATCC and grown as recommended. For TGFBI treatments, MMTV‐PyMT;*Tgfbi* ^Δ/Δ^ cells were grown on collagen‐coated plates and incubated with either 100 ng·mL^−1^ or 2 μg·mL^−1^ of exogenous TGFBI protein (a kind gift from Prof. J. Huelsken) for 72 h.

### 2.3 Tumour sphere assays

MMTV‐PyMT and C(3)TAg sphere cultures were prepared from fresh tumours. Tumour cells were obtained by tissue dissociation and plated on collagen‐coated plates overnight, trypsinised the next day, and plated in 150 μL of sphere media (DMEM/F12 with B27, 20 ng·mL^−1^ EGF, 20 ng·mL^−1^ FGF, 4 μg·mL^−1^ heparin, 1% penicillin/streptomycin) into 96‐well low attachment plates (Corning, NY, USA) at 1 × 10^4^ cells per well and at least three wells per tumour. For all other models, we plated 10^3^ cells per well. Spheres were counted after one week (7–10 days).

### 2.4 FACS analysis

For FACSorting experiments, tumour cells were obtained by enzymatic disaggregation as described above. Cells were then washed twice with PBS, strained through 70 μm nylon mesh strainers, stained with the appropriate antibodies for 30 min at 4 °C and sorted using either a FACSAria, a FACSAria III (BD Biosciences, Franklin Lakes, NJ, USA), or a MoFlo Astrios (Beckman Coulter, Brea, CA, USA). For FACS analysis, tumour cells were obtained by enzymatic disaggregation or trypsinised, washed and stained with the appropriate antibodies for 30 min at 4 °C. DAPI or 7‐AAD was used to discard dead cells. ALDH activity was tested using the AldeFluor assay kit (STEMCELL Technologies) as per the manufacturer's protocol. Briefly, cells were incubated with either the AldeFluor reagent alone or together with the inhibitor diethylaminobenzaldehyde (DEAB) for 30 min at 37 °C. Cells were then centrifuged, washed and immunophenotyped when required. Fluorescence was analysed using either a Cyan ADP (Dako‐Agilent, Santa Clara, CA, USA) or a MACSQuant (Miltenyi Biotec, Bergisch Gladbach, Germany) instrument. Data were processed and analysed using flowjo (Becton Dickinson, Franklin Lakes, NJ, USA).

### 2.5 MACS

For MACSorting experiments, tumour cells were obtained by enzymatic disaggregation, washed twice with PBS and strained through 70 μm nylon mesh strainers. MACS was then performed using the EasySep APC Positive Selection Kit (STEMCELL Technologies) following the manufacturer's instructions of. Briefly, FcR blocker was added to the sample and then incubated with a CD11b APC‐conjugated antibody. Next, the sample was incubated with the selection cocktail, followed by an incubation with immunomagnetic beads. CD11b^+^ cells were then isolated using an EasySep magnet (STEMCELL Technologies).

### 2.6 Mouse work

MMTV‐PyMT (FVB) mice were breed and housed in ventilated cages in the OHB mouse husbandry of the University of Fribourg. The *Tgfbi* ^∆/∆^ mouse was a kind gift by E. Wagner. For PyMT and C(3)TAg tumour cell transplantation to the 4th mammary fat pad or tail vein injection experiments, we used NODSCID‐Il2rg and immunocompetent FVB mice. The experiments involving 4T1 cell injections were done in immunocompetent BALB/c mice. Human breast cancer cells were injected into the 4th mammary fat pad or via tail vein in NODSCID‐Il2rg mice. Cells were trypsinised, resuspended in complete media and centrifuged at 370 ***g***. They were washed twice in PBS, counted and resuspended in PBS at the desired concentration for injection in PBS or Matrigel : PBS (1 : 3). For bone marrow transplantation experiments, female mice were anaesthetised with injection anaesthesia. Once the mice were asleep, they were placed on a warming pad, given eye moistening drops while limbs were fixed to a board with tape in the 50 cm stage. Mice were then irradiated at 220 kV, 20 mA and Al filtering using a dedicated X‐ray unit (Precision X‐Ray X‐RAD‐225) for a total dose of 13 Gy. Once finished, the mice were then transferred in their cage under IR light and observed till they recovered. In a period ranging from 6 to 48 h, the irradiated mice (recipients) were injected i.v. > 3 × 10^6^ bone marrow cells from donor mice. All the experiments involving mice were carried out in accordance with the Swiss Animal Welfare Regulations and were previously approved by the Cantonal Veterinary Service of the Canton Fribourg (2015_20_FR and 2017_26_FR).

### 2.7 Tail vein injections

Mice were warmed by placing the cage under an IR light bulb. One mouse at a time was placed in a tube rodent holder for tail vein injection with the tail outside of the tube. The tail was cleaned with 70% ethanol. IR light bulb was placed above the tail to cause the veins to dilate. MMTV‐PyMT tumour cells (5 × 10^5^ per mouse) were resuspended in PBS and injected very slowly in a 100 μL volume into one of the two tail veins using an insulin syringe (26G needle). The spot of injection was then compressed with a tissue to make sure the tail was not bleeding. Mice were returned to the cage and kept for observation for 15 min. Metastatic foci in the lungs were counted after 3–5 weeks using a Leica M125 stereomicroscope (Wetzlar,Germany).

### 2.8 Vasculature analysis and immunostainings

Immunostaining was performed on 4 μm thick paraffin sections using antigen retrieval for 20 min in boiling 10 mm citric acid, pH6.0. After blocking, we incubated the sections with the indicated antibodies overnight at 4 °C. We used secondary fluorescently labelled antibodies Alexa Fluor 488, 568 and 647 (Molecular Probes, Invitrogen), Cy3 (Jackson ImmunoResearch, West Grove, PA, USA) or HRP‐conjugated secondary antibodies. The breast cancer tissue array containing information on the molecular subtype was purchased from US Biomax (Derwood, MD, USA). The intensity of the IHC was evaluated independently by both authors and graded 1 to 4. To study hypoxia, we used pimonidazole (Hypoxyprobes). 60 mg·kg^−1^ pimonidazole was injected i.p. and was left to circulate for 1 h. For perfusion experiments, mice were given an intravenous injection of 100 μg of fluorescein isothiocyanate‐labelled tomato lectin (*Lycopersicon esculentum*; Vector Laboratories, Burlingame, CA, USA). After 10 min, the tissues were fixed with 4% PFA, and tumours were frozen in Tissue‐Tek optimum cutting temperature compound (Sakura, Torrance, CA, USA). To study vessel leakiness, mice were given an intravenous injection of 1 mg of 70‐kDa fluorescein isothiocyanate–dextran (Sigma). After 10 min, the tissues were fixed with 4% PFA, and tumours were frozen in Tissue‐Tek optimum cutting temperature compound. Mice were anaesthetised with 100 mg·kg^−1^ of ketamine and 10 mg·kg^−1^ of xylazine before injection of the reagents. Fluorescent images were taken with an automated upright microscope system DM5500 (Leica) or a LSM700 upright or inverted confocal microscope (Zeiss, Oberkochen, Germany). Light images were taken with an AX70 widefield microscope (Olympus, Shinjuku, Tokyo, Japan).

### 2.9 Western blot

Protein was extracted with complete RIPA buffer [20 mm Tris/HCl (pH 7.5), 150 mm NaCl, 1 mm Na2EDTA, 1 mm EGTA, 1% NP‐40, 1% sodium deoxycholate, 2.5 mm sodium pyrophosphate, 1 mm β‐glycerophosphate, 1 mm Na_3_VO_4_, 1 μg·mL^−1^ leupeptin; Cell Signaling], separated by electrophoresis, transferred to PVDF membranes (Millipore, Burlington, Massachusetts, USA), blocked with 5% BSA (Carl Roth, Karlsruhe, Germany) in 0.1% Tween 20 containing Tris‐buffered saline (TBST) and incubated overnight with primary antibodies. Immunoreactive bands were visualised using HRP‐conjugated secondary antibodies (Cell Signaling, Danvers, MA, USA, and Dako).

### 2.10 Lentiviral production

Lentiviral particles were produced in HEK293T cells by calcium phosphate precipitation. Briefly, 2 h prior to transfection, 11 × 10^6^ HEK293T cells/15 cm dish were incubated with DMEM + 10%FBS supplemented with 25 μm chloroquine (Sigma). Lentivirus was produced by cotransfection of HEK293T cells with the vectors of interest together with the pCMV‐dR8.74 and the pMD2G (VSVG). Next day, media was removed and replaced with fresh media containing 3 mm caffeine (Sigma). On the third day, the supernatant containing the viral particles was collected, ultracentrifuged for 2.5 h at 20 000 r.p.m. to concentrate the lentiviruses and used to infect cells or aliquoted and stored at −80 °C.

### 2.11 Real‐time PCR

RNA was prepared using the mini or micro RNA kit (Qiagen, Hilden, Germany) as per the manufacturer's instructions. cDNAs were generated using oligo‐T priming and the M‐MLV Reverse Transcriptase RNase H (–) Point Mutant (Promega, Madison, WI, USA). qPCR was performed in a StepOnePlus thermocycler (Applied Biosystems, Foster City, CA, USA) using the SYBR green PCR Master Mix (Kapa) and following the manufacturer's instructions. A list of primers used is shown in Table S1.

### 2.12 RNA sequencing

The RNA sequencing experiments were performed in the Swiss Integrative Center for Human Health (SICHH). All samples were first tested for integrity on a Fragment Analyser (ATTI) with the (DNF‐471) Standard Sensitivity RNA Analysis Kit (15 nt). Sequencing libraries were prepared and pooled using the TruSeq^®^ Stranded mRNA Library Prep kit (Illumina, San Diego, CA, USA) as instructed by the manufacturer. The sequencing was performed on a NextSeq 500 sequencer (Illumina), using the NextSeq 500/550 HT reagent kit v2 as instructed by the manufacturer. cutadapt version 1.16 was used to remove the adapter sequences from the reads and for quality trimming. All paired‐end reads were discarded if one of the reads was shorter than 20 bases.

The reads were then aligned against the mouse genome (assembly GRCm38/mm10) using star version 2.6.0a [20]. We acknowledge our use of the gene set enrichment analysis, gsea software and Molecular Database (MSigDB) [21].

### 2.13 Cibersort

cibersort is an algorithm designed to estimate the cell composition of a complex sample [22]. METABRIC data analyses were performed with 1000 permutations, the default signature matrix for immune cell subtypes (LM22), default statistical parameters and enabled quantile normalisation. Samples were filtered for *P* ≤ 0.05. The analysis of MMTV‐PyMT samples was performed using the signature matrix ImmuCC [23], with 1000 permutations and disabled quantile normalisation.

### 2.14 Statistics

The results were analysed using the graphpad prism software (San Diego, CA, USA). Means were compared with two‐tailed unpaired Student's *t*‐test. In case groups would not pass normality test (assessed using D'Agostino‐Pearson's omnibus normality test), samples were analysed with Mann–Whitney's nonparametric test. When comparing more than two variables, we performed one‐way analysis of variance. To isolate differences between groups, we performed the LSD test. In case groups would not pass normality, samples were analysed using the Kruskal–Wallis test. The METABRIC dataset [24], including clinical data and normalised gene expression, was retrieved through cbioportal [25]. Survival analyses were performed using the Mantel–Cox and the Gehan–Breslow–Wilcoxon tests. Cutpoints for survival analyses were determined using the application Evaluate cutpoints [26]. *P*‐values are indicated for each experiment. Limiting dilution assay data were analysed using ELDA (extreme limiting dilution assay) [27]. Experiments were done at least in triplicate. Error bars indicate standard deviation unless stated otherwise. Significant differences between experimental groups are indicated with asterisks as follows: *P < 0.05, **P < 0.01, ***P < 0.001 and ****P < 0.0001.

## 3 Results

### 3.1 TGFBI affects tumour‐initiating potential and metastasis in breast cancer

**Fig. 1:**
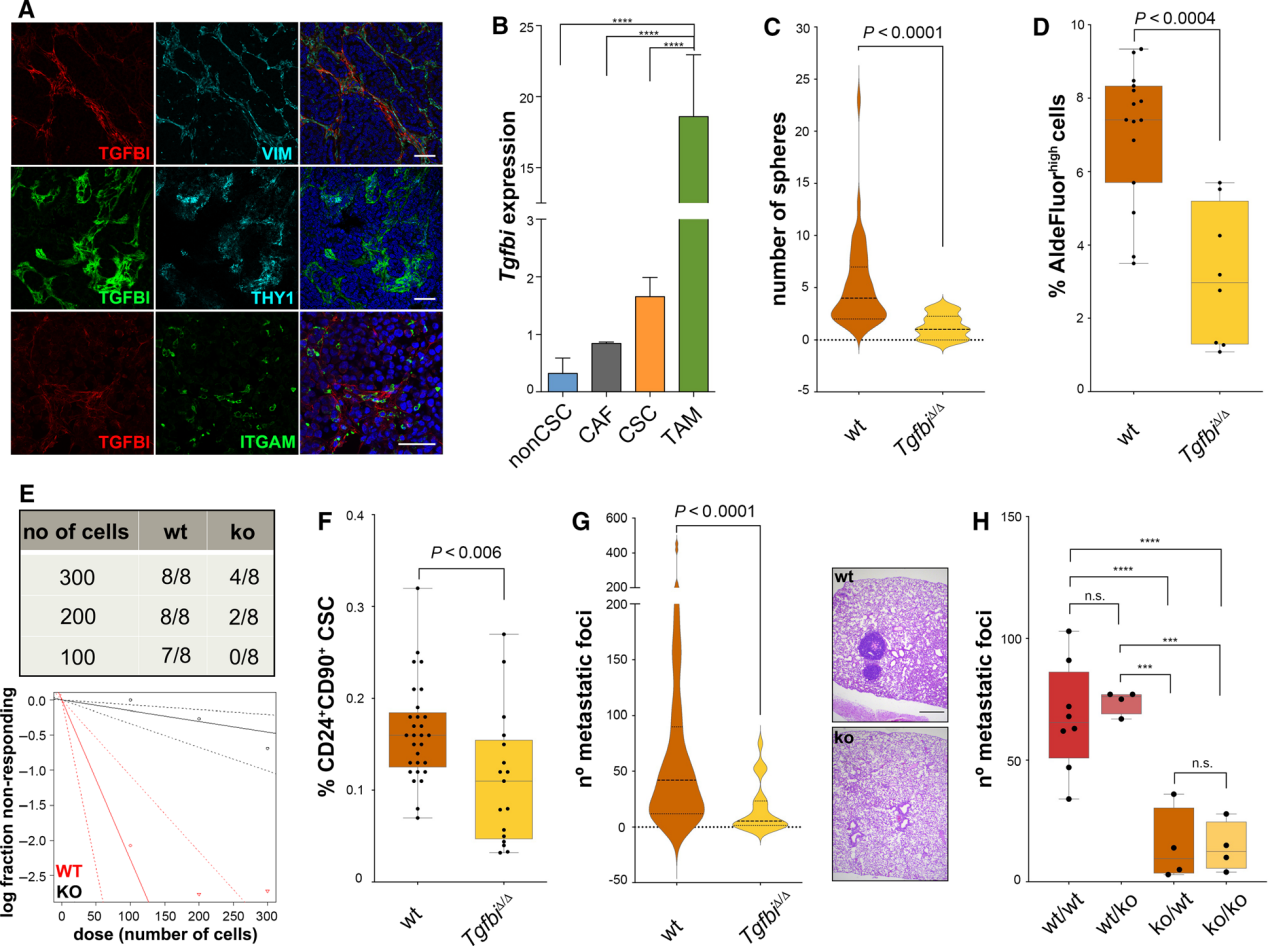
TGFBI and CSC potential. (A) Immunofluorescent staining for TGFBI, VIM, THY1 (CD90) and ITGAM (CD11b) in MMTV‐PyMT tumour sections. Scale bars, 50 μm. (B) qPCR analyses on FACS‐sorted Lin^−^CD24^+^CD90^−^ (nonCSC, *n* = 4), Lin^−^CD24^−^CD90^+^ (CAF, *n* = 2), and Lin^−^CD24^+^CD90^+^ (CSC, *n* = 4), or MACS‐sorted CD11b^+^ cells (TAM, *n* = 4) from fresh MMTV‐PyMT tumours showed differences in *Tgfbi* expression. Data were analysed using one‐way ANOVA followed by Fisher's LSD test, and are presented as mean and SD (*n* = 4 independent tumours); *Actb* was used as a housekeeping gene. (C) Tumour cells were obtained from fresh MMTV‐PyMT;*Tgfbi* ^+/+^ and MMTV‐PyMT;*Tgfbi* ^Δ/Δ^ tumours, grown overnight in collagen‐coated plates, and seeded as spheres (10^4^ cells/well). Spheres were counted 10 days later. Data were analysed by Mann–Whitney test and are presented as a violin plot showing the median and the quartiles (*n* = 4 wt, *n* = 2 ko independent tumours). (D) Cells from fresh MMTV‐PyMT;*Tgfbi* ^+/+^ and MMTV‐PyMT;*Tgfbi* ^Δ/Δ^ tumours were analysed by FACS using the AldeFluor assay. Data were analysed by Mann–Whitney test (*n* = 15 wt, *n* = 8 ko). (E) MMTV‐PyMT;*Tgfbi* ^+/+^ and MMTV‐PyMT;*Tgfbi* ^Δ/Δ^ tumours were digested; tumour cells were counted and injected orthotopically in limiting dilution assays in FVB/N mice. The presence or absence of tumours was evaluated for a maximum of 3 months after injection. Data were analysed using ELDA (*P* = 1.61e^−08^). (F) Cells from fresh MMTV‐PyMT;*Tgfbi* ^+/+^ and MMTV‐PyMT;*Tgfbi* ^Δ/Δ^ tumours were analysed by FACS for their expression of CD24 and CD90. Data were analysed by Mann–Whitney test (*n* = 29 wt, *n* = 17 ko). (G) Number of metastatic foci in the lungs of MMTV‐PyMT;*Tgfbi* ^+/+^ and MMTV‐PyMT;*Tgfbi* ^Δ/Δ^ mice. Data were analysed by Mann–Whitney test and are presented as a violin plot showing the median and the quartiles (*n* = 55 wt, *n* = 17 ko). Representative haematoxylin–eosin staining of MMTV‐PyMT;*Tgfbi* ^+/+^ and MMTV‐PyMT;*Tgfbi* ^Δ/Δ^ lungs (scale bar 500 μm). (H) Number of metastatic foci in the lungs of wt and ko FVB/N mice injected with 5 × 10^5^ MMTV‐PyMT;*Tgfbi* ^+/+^ and MMTV‐PyMT;*Tgfbi* ^Δ/Δ^ tumour cells. Data were analysed using one‐way ANOVA followed by Fisher's LSD test (*n* = 8 wt/wt, *n* = 4 wt/ko, *n* = 4 ko/wt, *n* = 4 ko/ko). CSC, cancer stem cell; CAF, cancer‐associated fibroblast; TAM, tumour‐associated macrophage. ***P < 0.001; ****P < 0.0001; n.s., not significant.

We first analysed MMTV‐PyMT mammary gland tumours for the expression of TGFBI. As shown in Fig. 1A, immunostainings revealed that TGFBI is mainly expressed in the stromal compartment, close to regions containing vimentin‐positive cells and CD90^+^ cells [4]. Since it has been shown that TGFBI can bind and recruit macrophages through the integrin ITGAM (CD11b) [28], we performed costainings and showed that indeed TGFBI is found in close contact to CD11b^+^ cells (Fig. 1A). To identify and quantify the cellular sources of TGFBI, we next isolated by FACS and MACS different cell populations from these tumours and analyse them by qPCR. We found that CD11b^+^ cells expressed the highest levels of *Tgfbi*, followed by CD24^+^CD90^+^ CSCs and CD24^−^CD90^+^ stromal cells (Fig. 1B). Of note, up to 90% of CD11b^+^ MACSorted cells were also F4/80^+^, indicating that they are macrophages (Fig. S1a). The pattern of expression is similar in other murine breast cancer models (Fig. S1b). To understand the biological effects that TGFBI exerts on CSCs, we crossed a *Tgfbi* knockout mouse with the MMTV‐PyMT model (Fig. S1c–e). We first tested the ability of these tumours to form mammospheres and observed that MMTV‐PyMT;*Tgfbi* ^Δ/Δ^ tumours possess less sphere‐forming cells (Fig. 1C). We had previously shown that MMTV‐PyMT derived spheres are formed by tumour‐initiating ALDH^high^ cells and contain a mixture of cell types [6]. Indeed, FACS analyses revealed that MMTV‐PyMT;*Tgfbi* ^Δ/Δ^ tumours have reduced numbers of ALDH^high^ cells and express lower levels of *Aldh1a3* (Fig. 1D and Fig. S1f,g). Accordingly, tumour cells derived from TGFBI‐deficient mice have 37 times less tumour‐initiating capacity in limiting dilution assays (Fig. 1E). In addition, MMTV‐PyMT;*Tgfbi* ^Δ/Δ^ tumours also showed a significant reduction in metastatic Lin^−^CD24^+^CD90^+^ cells (Fig. 1F) and consequently seeded less lung metastases (Fig. 1G). These results were confirmed by lung metastasis assays, which demonstrated that the metastatic capacity of the tumour cells is determined by their origin, regardless of the genotype of the host in which they were injected (Fig. 1H). However, sphere, tumour and metastasis formation were not significantly decreased or increased by either knocking out or overexpressing *Tgfbi* in breast cancer cells (Fig. S2). These results indicate that TGFBI‐driven microenvironmental changes in the primary tumour influence the CSC phenotype. Taken together, these data suggest that TGFBI in the ECM is an important driver of tumour‐ and metastasis‐initiating capacity in breast cancer.

### 3.2 TGFBI depletion normalises the tumour vasculature and reduces tumour hypoxia

**Fig. 2:**
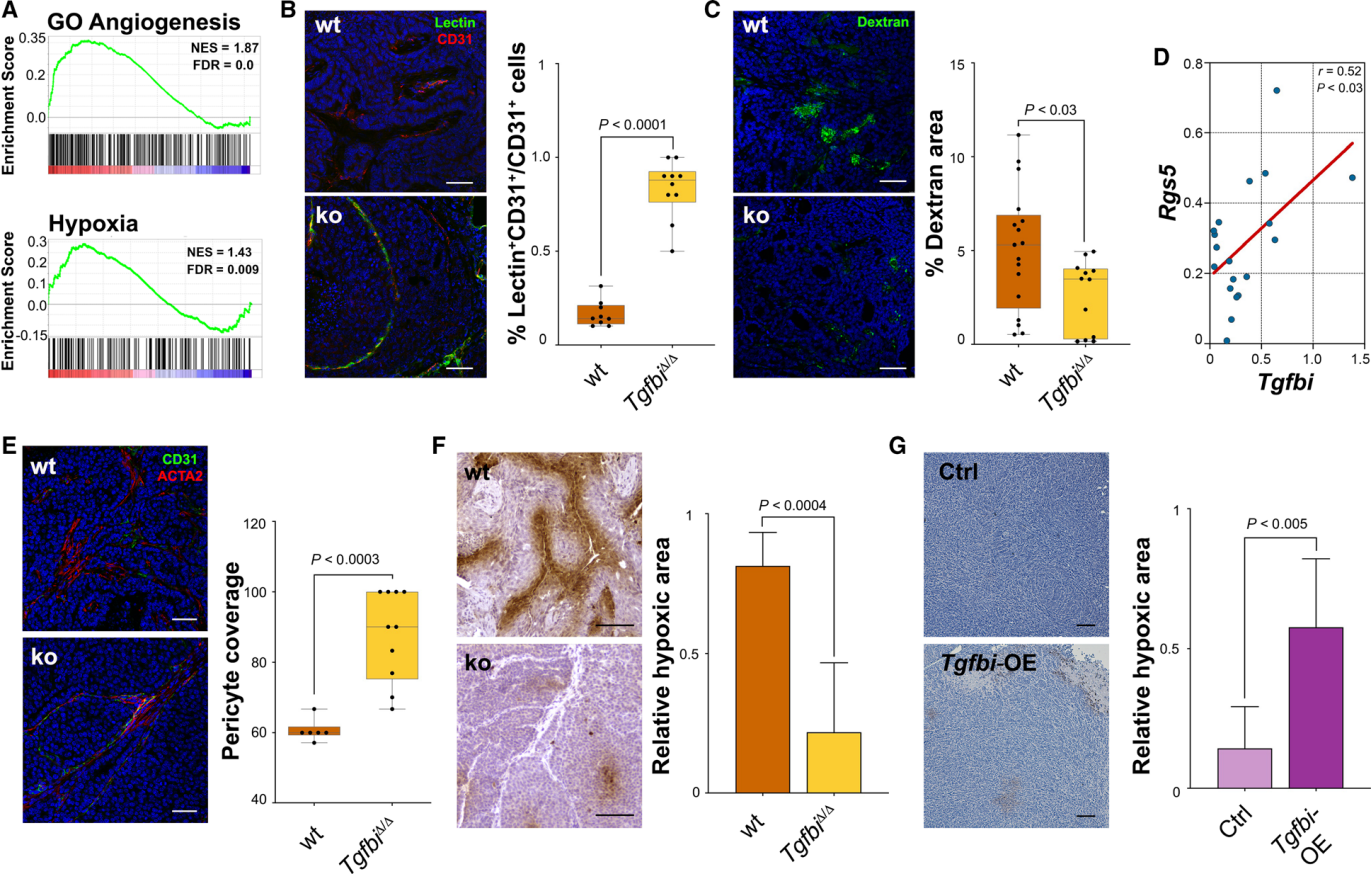
TGFBI depletion normalises the tumour vasculature. (A) Enrichment score analyses of RNAseq data obtained from MMTV‐PyMT;*Tgfbi* ^+/+^ and MMTV‐PyMT;*Tgfbi* ^Δ/Δ^ tumours (*n* = 5) for GO angiogenesis and hallmark hypoxia. (B) MMTV‐PyMT;*Tgfbi* ^+/+^ and MMTV‐PyMT;*Tgfbi* ^Δ/Δ^ mice were injected via tail vein with 100 μg of tomato lectin, and tumours were embedded in OCT and costained with CD31 (scale bar 100 μm). Data were analysed by unpaired *t*‐test. (C) MMTV‐PyMT;*Tgfbi* ^+/+^ and MMTV‐PyMT;*Tgfbi* ^Δ/Δ^ mice were injected via tail vein with 50 μg of 70 kDa FITC‐labelled dextran, and tumours were embedded in OCT (scale bar 100 μm). Data were analysed by unpaired *t*‐test. (D) *Tgfbi* and *Rgs5* expression correlation in MMTV‐PyMT tumours. The expression of both genes was determined by qPCR. The correlation was estimated by Pearson's *r* coefficient (*n* = 19). *Rplp0* was used as a housekeeping gene. (E) Immunofluorescent staining of MMTV‐PyMT;*Tgfbi* ^+/+^ and MMTV‐PyMT;*Tgfbi* ^Δ/Δ^ tumours with CD31 and ACTA2. Data were analysed by unpaired *t*‐test. (F) PIMO staining of MMTV‐PyMT;*Tgfbi* ^+/+^ and MMTV‐PyMT;*Tgfbi* ^Δ/Δ^ tumours and quantification of stained area (scale bar 100 μm). Data were analysed by unpaired *t*‐test on two independent tumours, and are presented as mean and SD. (G) PIMO staining of MDA‐MB‐453 control and *Tgfbi*‐overexpressing tumours and quantification of stained area (scale bar 100 μm). Data were analysed by unpaired *t*‐test, and are presented as mean and SD (*n* = 3 ctrl tumours, *n* = 4 overexpression tumours). PIMO, pimonidazole.

To gain a better understanding of the pathways affected by the deletion of TGFBI, we performed RNA sequencing of wt and ko tumours. GSEA analyses showed that both angiogenesis and hypoxia were significantly reduced in ko tumours (Fig. 2A, and Figs S3 and S4). In view of these results, we next aimed at understanding whether depletion of TGFBI impacts the tumour vasculature functionality. We first determined vascular perfusion and leakiness performing tomato lectin and 70 kDa FITC‐labelled dextran intravenous injections. The former stains all vessels with an active perfusion and the latter seeps out of the vasculature in ruptured vessels. Our results indicate that TGFBI depletion leads to increased perfusion (Fig. 2B) and reduced vascular leakage (Fig. 2C). Interestingly, we observed a positive correlation of *Tgfbi* with regulator of G protein signalling 5 (*Rgs5*), a gene whose expression has been linked to the formation of aberrant vasculature in tumours (Fig. 2D and Fig. S5) [29]. Furthermore, TGFBI loss led to increased and more structured pericyte coverage (Fig. 2E). As a consequence of improved perfusion, *Tgfbi* ko tumours exhibit less hypoxic areas (Fig. 2F) and consequently, decreased features of epithelial‐to‐mesenchymal (EMT) transition (Fig. S6a). To uncouple potential direct effects of TGFBI on cancer cells from those on the tumour vasculature, we added exogenous TGFBI to MMTV‐PyMT;*Tgfbi* ^Δ/Δ^ cells in culture. However, adding TGFBI did not trigger EMT (Fig. S6b), which again indicates that decreased CSC numbers and metastases are mainly the result of TGFBI's microenvironmental effects. *In silico* analyses using the METABRIC cohort confirmed that the expression of *TGFBI* in human breast tumours positively correlates with the expression of many hypoxia‐related genes (Fig. S7). Likewise, *Tgfbi* overexpression in breast cancer cells increased tumour hypoxia (Fig. 2G). Overall, these results indicate that TGFBI promotes aberrant angiogenesis and increases tumour hypoxia in breast cancer.

### 3.3 TGFBI levels determine tumour hypoxia

**Fig. 3:**
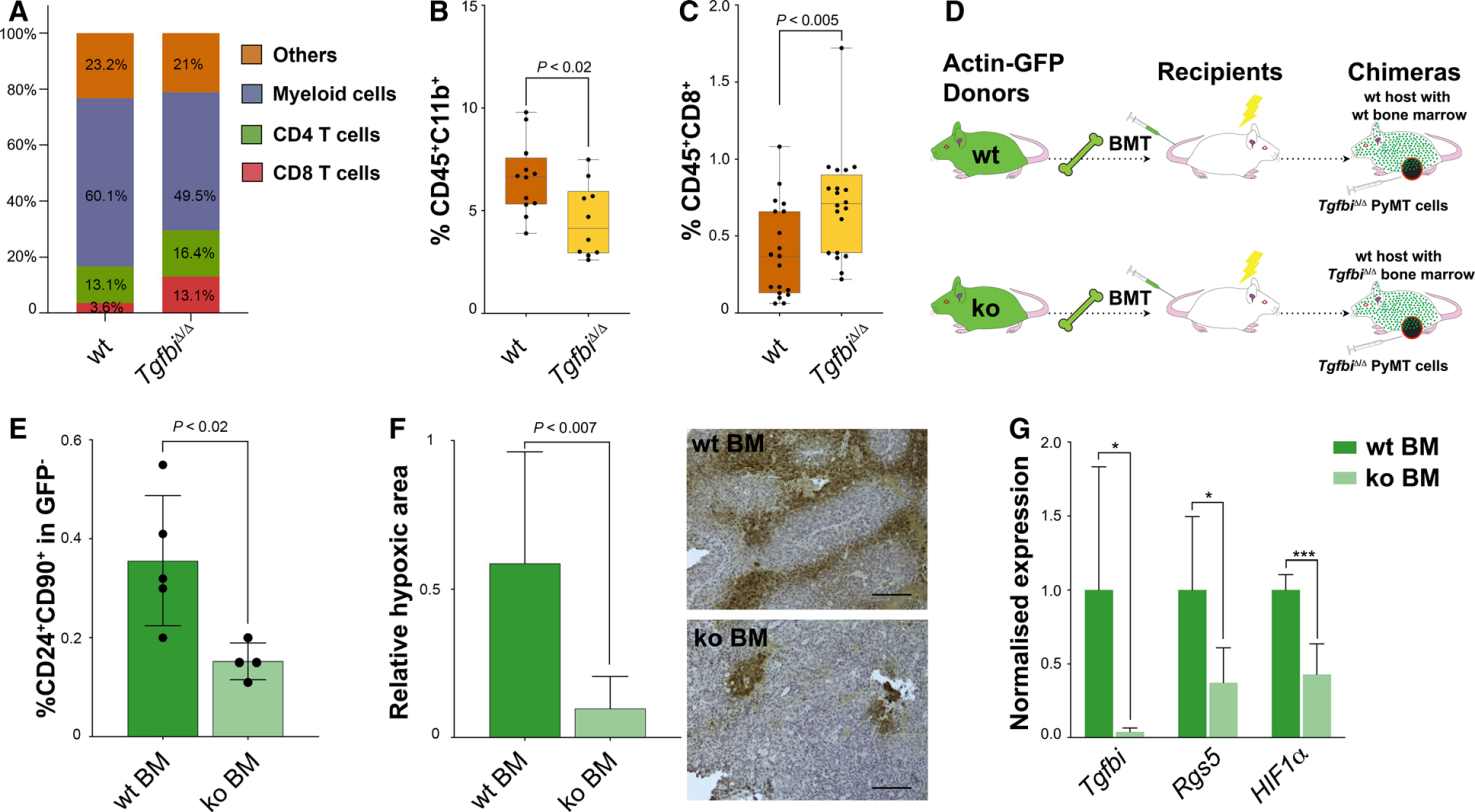
TGFBI levels determine tumour hypoxia. (A) cibersort analysis of MMTV‐PyMT;*Tgfbi* ^+/+^ and MMTV‐PyMT;*Tgfbi* ^Δ/Δ^. (B) The presence of CD45^+^CD11b^+^ cells was determined by FACS in fresh MMTV‐PyMT;*Tgfbi* ^+/+^ and MMTV‐PyMT;*Tgfbi* ^Δ/Δ^ tumours. Data were analysed by unpaired *t*‐test (*n* = 12 wt, *n* = 10 ko). (C) FACS analysis of CD8^+^ T cells in fresh MMTV‐PyMT;*Tgfbi* ^+/+^ and MMTV‐PyMT;*Tgfbi* ^Δ/Δ^ tumours. Data were analysed by Mann–Whitney's test (*n* = 19 wt, *n* = 20 ko). (D) Bone marrow transplantation experimental scheme. GFP^+^ bone marrows from *Tgfbi* ^+/+^ or *Tgfbi* ^Δ/Δ^ mice were transplanted into lethally irradiated wild‐type hosts. The resultant chimeras were orthotopically injected with MMTV‐PyMT;*Tgfbi* ^Δ/Δ^ tumour cells. The content of CD24^+^CD90^+^ (E) in these tumours was analysed by FACS. Data were analysed by unpaired *t*‐test, and are presented as mean and SD (*n* = 5 wt, *n* = 4 ko). (F) PIMO staining in *Tgfbi* ^+/+^ and *Tgfbi* ^Δ/Δ^ bone marrow transplanted mice tumours and quantification of stained area (scale bar 100 μm). Data were analysed by unpaired *t*‐test, and are presented as mean and SD (*n* = 3). (G) *Tgfbi*, *Rgs5,* and *Hif1α* qPCR on pulverised tumour material from *Tgfbi* ^Δ/Δ^ chimeras. Data were analysed by unpaired *t*‐test, and are presented as mean and SD (*n* = 5). *Rplp0* was used as a housekeeping gene. BM, bone marrow; BMT, bone marrow transplantation; PIMO, pimonidazole. *P < 0.05; ***P < 0.001; n.s., not significant.

In order to evaluate the effects of TGFBI deletion in the tumour immune landscape, we first used the cibersort software [22, 23]. The analysis of our tumour samples revealed significant differences in the myeloid and the T‐cell compartments (Fig. 3A), with *Tgfbi* ^Δ/Δ^ tumours having less myeloid cells and more T lymphocytes. These findings were confirmed by immunophenotyping the MMTV‐PyMT tumours by FACS (Fig. 3B,C). Since we had previously found that in MMTV‐PyMT tumours macrophages secreted high levels of TGFBI, we argued that it should be possible to induce tumour hypoxia by transferring wt macrophages into lethally irradiated mice bearing *Tgfbi* ^Δ/Δ^ tumours. Therefore, we performed wt and ko bone marrow transplants (BMT) into wt mice, which were afterwards orthotopically injected with *Tgfbi* ^Δ/Δ^ tumour cells (Fig. 3D). *Tgfbi*‐deficient tumours grown in wt mice transplanted with ko bone marrow cells showed a decreased number of CSCs (Fig. 3E). In addition, these tumours were less hypoxic and expressed lower levels of *Rgs5* (Fig. 3F,G). These results confirm that CD11b^+^ cells are a main source of TGFBI in MMTV‐PyMT tumours, and they indicate that TGFBI levels associate with tumour angiogenesis and hypoxia.

### 3.4 TGFBI is associated with poor prognosis

**Fig. 4:**
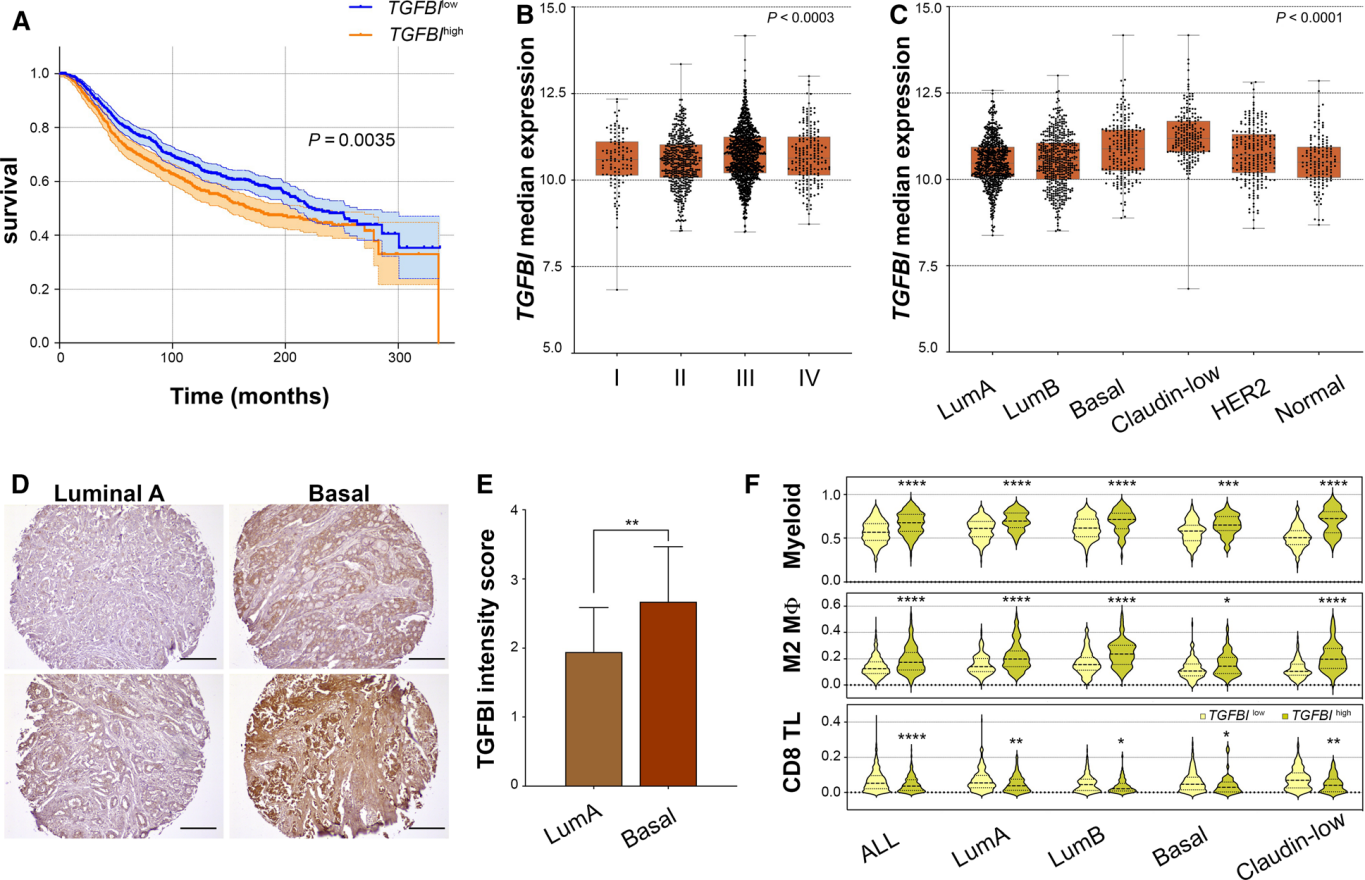
TGFBI is associated with poor prognosis. (A) Survival curves of breast cancer patients in the METABRIC cohort classified according to the expression of *TGFBI* (*n*_high_ = 620; *n*_low_ = 803). Patients were stratified using the application *Evaluate cutpoints*, and the survival curves were compared using the log‐rank (Mantel–Cox) test. Only ‘living’ and ‘died of disease’ patients were included in the analysis. (B) *TGFBI* expression in the METABRIC cohort classified according to the Nottingham prognostic index (*n*_I_ = 108, *n*_II_ = 452, *n*_III_ = 1064, *n*_IV_ = 191). Results were analysed using the Kruskal–Wallis test. (C) *TGFBI* expression of breast cancer patients in the METABRIC cohort classified according to the molecular subtype (*n*_LumA_ = 679, *n*_LumB_ = 461, *n*_Basal_ = 199, *n*_Claudin‐low_ = 199, *n*_HER2_ = 220, *n*_Normal_ = 140). Results were analysed using the Kruskal–Wallis test followed by Dunn's multiple comparison test (LumA vs. LumB n.s., LumA vs. Basal *P* < 0.0001, LumA vs. Claudin‐low *P* < 0.0001, LumA vs. HER2 *P* < 0.0003, LumA vs. Normal n.s., LumB vs. Basal *P* < 0.0001, LumB vs. Claudin‐low *P* < 0.0001, LumB vs. HER2 *P* < 0.02, LumB vs. Normal n.s., Basal vs. Claudin‐low *P* < 0.0001, Basal vs. HER2 n.s., Basal vs. Normal *P* < 0.002, Claudin‐low vs. HER2 *P* < 0.0001, Claudin‐low vs. Normal *P* < 0.0001, HER2 vs. Normal n.s.) (D) Representative immunohistochemistry stainings for TGFBI in luminal A and basal‐like human breast tumours. (E) TGFBI IHC scoring according to the staining's intensity. Data was analysed using an unpaired *t*‐test, and are presented as mean and SD (*n* = 18 luminal A tumours, *n* = 22 basal‐like tumours). (F) Relative myeloid cell, M2 macrophage, and CD8 T‐cell content of *TGFBI* ^l^ ^ow^ (< 75th percentile) and *TGFBI* ^high^ tumours (≥ 75th percentile) in METABRIC tumours unclassified and classified by molecular subtype. The relative cell content estimation was calculated with cibersort. The data were analysed using either unpaired Student's *t*‐tests or Mann–Whitney's nonparametric tests, and they are presented as violin plots showing the median and the quartiles. *P < 0.05; **P < 0.01; ***P < 0.001; ****P < 0.0001; n.s., not significant.

In order to understand the relevance of TGFBI in human breast cancer, we performed *in silico* analysis using the METABRIC cohort [30] and found that TGFBI predicts poor prognosis (Fig. 4A). Interestingly, in patients undergoing chemotherapy, low TGFBI expression levels predict better prognosis (Fig. S8), supporting our experimental data on vessel normalisation, which had previously been linked to enhanced therapeutic efficacy [31, 32]. Moreover, its expression increases as a function of the Nottingham prognostic index (NPI, Fig. 4B), especially between patients with NPI ≤ 3.4 and NPI > 3.4. We next compared the expression of *TGFBI* in the different molecular subtypes of the METABRIC database and found that basal and claudin‐low tumours, which are known to contain high percentages of CSCs [33, 34], have the highest expression levels (Fig. 4C). We further confirmed these results by IHC on a panel of human breast cancer tissue samples. Our results indicate that basal tumours show higher TGFBI staining scores compared to luminal A tumours (Fig. 4D,E). We then estimated the abundance of different immune cell types in tumours expressing high or low levels of *TGFBI* in the METABRIC cohort using cibersort. As shown in Fig. 4F, the estimated relative content of myeloid cells in general, and M2 macrophages in particular, positively associates with *TGFBI* expression, while that of CD8^+^ T cells is negatively linked with *TGFBI* expression. Likewise, when every subtype was subdivided into high and low expressers, we found that *TGFBI* ^high^ tumours within the same molecular subtype contain more myeloid cells and M2 macrophages, but less CD8^+^ T cells (Fig. 4F), which agrees with our experimental data. Furthermore, GSEA analyses confirmed that *TGFBI* ^high^ tumours display molecular signatures of increased angiogenesis, hypoxia and EMT (Fig. S9). Taken together, these results indicate that *TGFBI* expression predicts tumour aggressiveness and poorer outcomes in human breast cancer, and it is associated with higher macrophage infiltration and angiogenesis.

## 4 Discussion

Aberrant angiogenesis and hypoxia influence each other by creating a positive forward loop that promotes tumour progression. Increased hypoxia exerts severe metabolic constraints on tumour cells and is a major driver that maintains and expands CSCs [13, 35, 36, 37]. Despite its promising potential, antiangiogenic therapies have failed to produce consistent, long‐lasting effects in mouse models and human patients [38]. In fact, reducing the tumour vasculature inevitably results in increased hypoxia, which in turn may increase CSC expansion and tumour chemoresistance [39]. Tumour vessel normalisation has several potential advantages, which include the improvement of drug delivery and the reduction of metastatic spread through the generation of a proper, well‐organised layer of pericytes around the vessels [31, 32, 40, 41, 42]. However, the molecular mechanisms controlling this reversal of the structure and function of the tumour vasculature are yet unclear. In the present study, we show that deletion of the matricellular protein TGFBI is sufficient to normalise the tumour vasculature and mitigate hypoxia. The role of TGFBI in cancer is still controversial and seems to be context‐dependent. For instance, TGFBI has been suggested to have tumour suppressive activities in mesothelioma, breast, and lung cancer cells [43, 44]. In addition, Zhang *et al*. [45] reported that homozygous null deletions of *Tgfbi* in mice result in increased frequency of spontaneous tumours and increased predisposition to cancer induction. Conversely, in colon cancer, TGFBI has been shown to favour extravasation, which in turn promotes metastasis [46]. In melanoma cells, TGFBI plays an anti‐adhesive role and its knockdown decreases tumour growth and invasion [47]. Likewise, in prostate cancer, TGFBI contributes to tumour progression [48]. Finally, recent work by Costanza *et al*. [49] showed that TGFBI predicts poor prognosis in pancreatic adenocarcinoma patients and promotes pancreatic cancer cell migration. Our results in breast cancer also suggest a tumour‐promoting role of TGFBI, but they indicate that modulating TGFBI in breast cancer cells does not directly impact the CSC phenotype and therefore the metastatic potential of tumour cells. However, using a *Tgfbi* straight knockout model, we found that the overall expression levels of TGFBI in a given tumour will determine the extent of hypoxia, EMT, and ultimately the CSC content and metastatic potential of the tumour. The differences observed between our study and previously published data might be due to methodological differences, in particular the fact that we focused on the role of TGFBI in the tumour microenvironment using a knockout mouse [43, 44]. We found that in the MMTV‐PyMT model, TGFBI is expressed mostly by macrophages, mesenchymal CSCs and stromal cells. Interestingly, Martinez *et al*. compared the transcriptional profiles of M1 vs M2 macrophages and found that *TGFBI* is expressed at higher levels in the latter [50], which are known to contribute to tumour angiogenesis [51]. Indeed, our analyses reveal that there is a positive association between *TGFBI* expression and M2 macrophage content in human breast tumours. Moreover, the levels of expression of *TGFBI* in human breast tumours significantly correlate with their molecular subtype. Interestingly, those subtypes enriched in mesenchymal CSCs [52] express higher levels of TGFBI, which is in agreement with our experimental findings. The mechanism by which TGFBI regulates tumour hypoxia in breast cancer and whether this effect is direct or indirect remains unclear. A number of nonexclusive factors may explain the effects that we observed. For instance, TGFBI is known to interact not only with several integrins [28, 46, 53, 54, 55, 56, 57, 58], but also with other components of the ECM, such as collagens, fibronectin, laminin, periostin and proteoglycans [59, 60, 61, 62, 63]. The reorganisation of the extracellular matrix and the initiation of integrin and focal adhesion kinase (FAK) signalling have direct implications for the regulation of angiogenesis and hypoxia [12, 64]. However, further work is needed to explore the interactions of TGFBI with endothelial cells and its potential role in macrophage biology and vascular maturation.

## 5 Conclusions

In summary, our study reveals a new biological role for the matricellular protein TGFBI in breast cancer. Our results indicate that TGFBI, which we found secreted by macrophages, mesenchymal tumour cells and CAFs, is a crucial player regulating breast tumour‐ and metastasis‐initiating potential through the modulation of the tumour microenvironment and hypoxia, and emphasise the importance of the extracellular matrix on breast cancer progression and metastasis. Taken together, our findings suggest that TGFBI may be used as a prognostic factor in breast cancer and open potential new opportunities for combinatorial therapies.

## Conflict of interest

The authors declare no conflict of interest.

## Author contributions

AS‐M conceptualized the study. AS‐M and FF contributed to methodology. FF and AS‐M involved in investigation. FF and AS‐M performed formal analysis. AS‐M provided resources. AS‐M and FF wrote the original draft of the manuscript. AS‐M and FF wrote, reviewed and edited the manuscript. AS‐M acquired funding. AS‐M supervised the study. All authors read and approved the final manuscript.

## Supporting information


**Fig. S1.** CD11b+ MACS, TGFBI IHC, *Tgfbi* knockout mouse model and ALDH in MMTV‐PyMT;*Tgfbi* ^Δ/Δ^ tumours.
**Fig. S2.** TGFBI genetic modulation in C(3)TAg and MDA‐MB‐453 cells.
**Fig. S3.** Heatmap of angiogenesis and hypoxia pathway genes regulated upon *Tgfbi* deletion.
**Fig. S4.** List of differentially expressed genes in MMTV‐PyMT;*Tgfbi* ^Δ/Δ^ tumours.
**Fig. S5.** Correlation between *Tgfbi* and *Rgs5* expression in 4T1 and MDA‐MB‐453 tumours.
**Fig. S6.** TGFBI and EMT.
**Fig. S7.** Correlation between *TGFBI* and hypoxia‐related genes in the METABRIC cohort.
**Fig. S8.** Survival curves for METABRIC patients who underwent chemotherapy stratified for *TGFBI* expression.
**Fig. S9.** GSEA analyses in the METABRIC cohort.
**Table S1.** List of primers used for qPCR.

## Data Availability

All data generated and analysed during this study are included in this manuscript and its supplementary files.
