# Supplementary material for: TGFBI modulates tumour hypoxia and promotes breast cancer metastasis
Source: Mol Oncol. 2020 Nov 5;14(12):3198–210. doi: 10.1002/1878-0261.12828 (PMC7718944; doi:10.1002/1878-0261.12828)
Supplement: Supplementary file 1 — Fig. S1. CD11b+ MACS, TGFBI IHC, Tgfbi knockout mouse model and ALDH in MMTV‐PyMT;Tgfbi Δ/Δ tumours. Fig. S2. TGFBI genetic modulation in C(3)TAg and MDA‐MB‐453 cells. Fig. S3. Heatmap of angiogenesis and hypoxia pathway genes regulated upon Tgfbi deletion. Fig. S4. List of differentially expressed genes in MMTV‐PyMT;Tgfbi Δ/Δ tumours. Fig. S5. Correlation between Tgfbi and Rgs5 expression in 4T1 and MDA‐MB‐453 tumours. Fig. S6. TGFBI and EMT. Fig. S7. Correlation between TGFBI and hypoxia‐related genes in the METABRIC cohort. Fig. S8. Survival curves for METABRIC patients who underwent chemotherapy stratified for TGFBI expression. Fig. S9. GSEA analyses in the METABRIC cohort. Table S1. List of primers used for qPCR. [file MOL2-14-3198-s001.pdf]

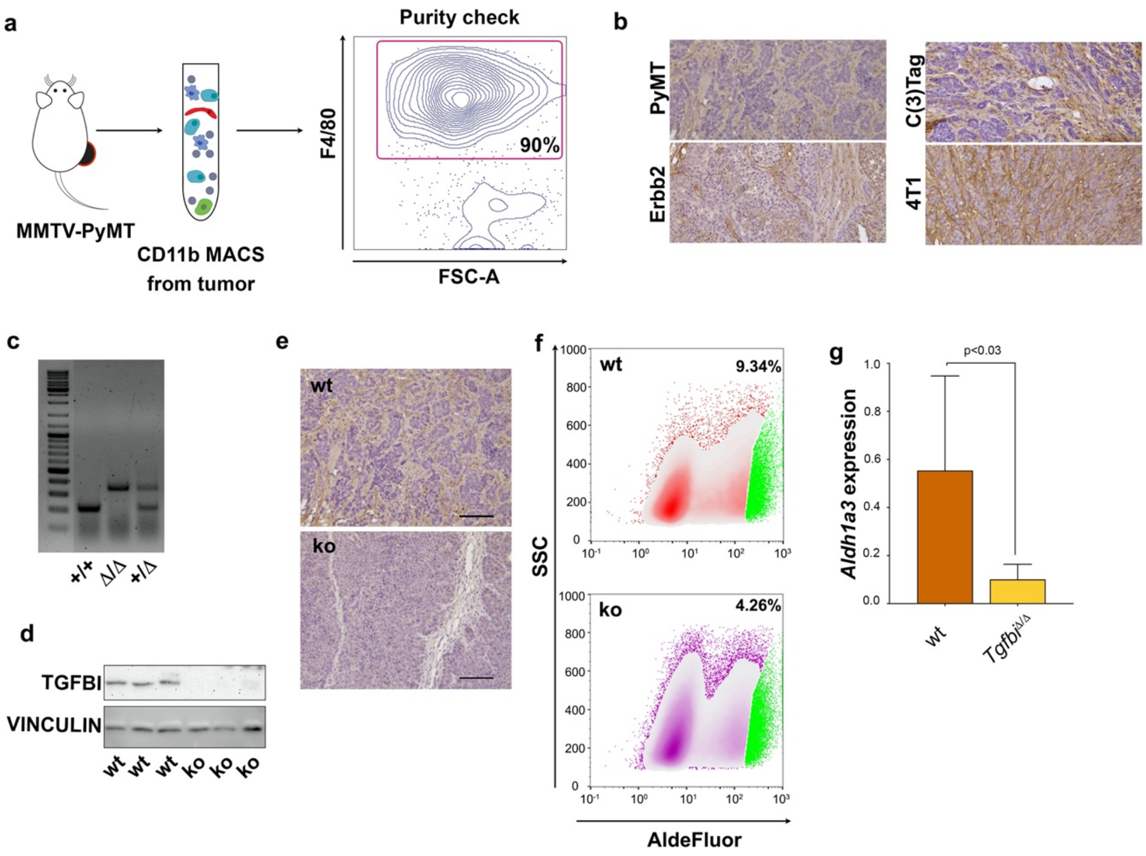

Supplemental File 1

Supplemental File 1: (a) CD11b^{+} cells from fresh MMTV-PyMT tumours were sorted by MACS and reanalysed by FACS for their expression of F4/80. (b) TGFBI immunohistochemistry of MMTV-PyMT, MMTV-Erbb2, C(3)TAg, and 4T1 tumours. (c) Genotyping PCR of *Tgfbi*$^{+/+}$, *Tgfbi*$^{\Delta/\Delta}$ and *Tgfbi*$^{\Delta/+}$ mice. (d) Western blot for TGFBI and vinculin (loading control) for 3 MMTV-PyMT;*Tgfbi*$^{+/+}$ and 3 MMTV-PyMT;*Tgfbi*$^{\Delta/\Delta}$ tumours. (e) Representative TGFBI IHC of a MMTV-PyMT;*Tgfbi*$^{+/+}$ and a MMTV-PyMT;*Tgfbi*$^{\Delta/\Delta}$ tumour. (f) Representative AldeFluor analysis FACS analysis of MMTV-PyMT;*Tgfbi*$^{+/+}$ and MMTV-PyMT;*Tgfbi*$^{\Delta/\Delta}$ tumours. (g) MMTV-PyMT;*Tgfbi*$^{+/+}$ and MMTV-PyMT;*Tgfbi*$^{\Delta/\Delta}$ tumours were pulverised, their RNA was extracted, subjected to reverse transcription and analysed by qPCR for the expression of *Aldh1a3*. Data were analysed by unpaired t test (n=4 wt, n=5 ko). *Rplp0* was used as a housekeeping gene.

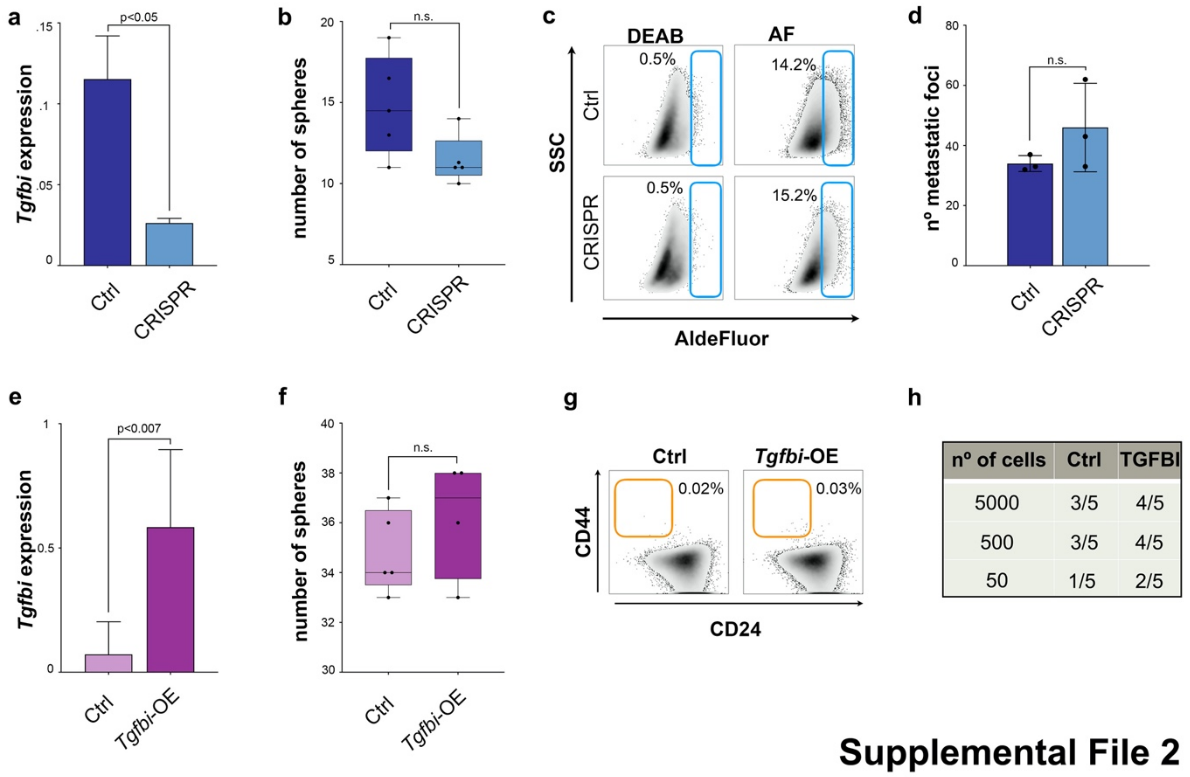

Supplemental File 2

Supplemental File 2: (a) C(3)TAg cells were infected with lentiviruses containing a CRISPR-*Tgfbi*, cells were selected and tested for expression of *Tgfbi* by qPCR. Data were analysed by unpaired t test (n=2). *Rplp0* was used as a housekeeping gene. (b) C(3)TAg cells wt or ko for TGFBI were seeded as spheres (10^3 cells/ well). Spheres were counted 10 days later. Data were analysed by unpaired t test (n =5 wt, n=5 ko). (c) C(3)TAg cells wt or ko for TGFBI were analysed by FACS using the AldeFluor assay. (d) $5x10^5$ C(3)TAg cells wt or ko for TGFBI were injected into NSG mice via tail vein. Metastatic foci were counted 4 weeks later. Data were analysed by unpaired t test (n=3). (e) *Tgfbi* qPCR in MDA-MB-453 cells infected with control or *Tgfbi* lentiviruses. *SNRPD3* was used as a housekeeping gene. (f) MDA-MB-453 cells overexpressing *Tgfbi* and their respective controls were seeded as spheres (10^3 cells/ well). Spheres were counted 10 days later. Data were analysed by unpaired t test (n =5 wt, n=4 *Tgfbi*-OE). (g) MDA-MB-453 cells overexpressing *Tgfbi* and their respective controls were analysed by FACS for the presence of $CD24^{low}CD44^{high}$ cells. (h) MDA-MB-453 cells overexpressing *Tgfbi* and their respective controls were trypsinised, counted, and injected orthotopically in limiting dilution assays in NSG mice. The presence or absence of tumours was evaluated for a maximum of 3 months after injection. Data were analysed using ELDA (*P*=0.14).

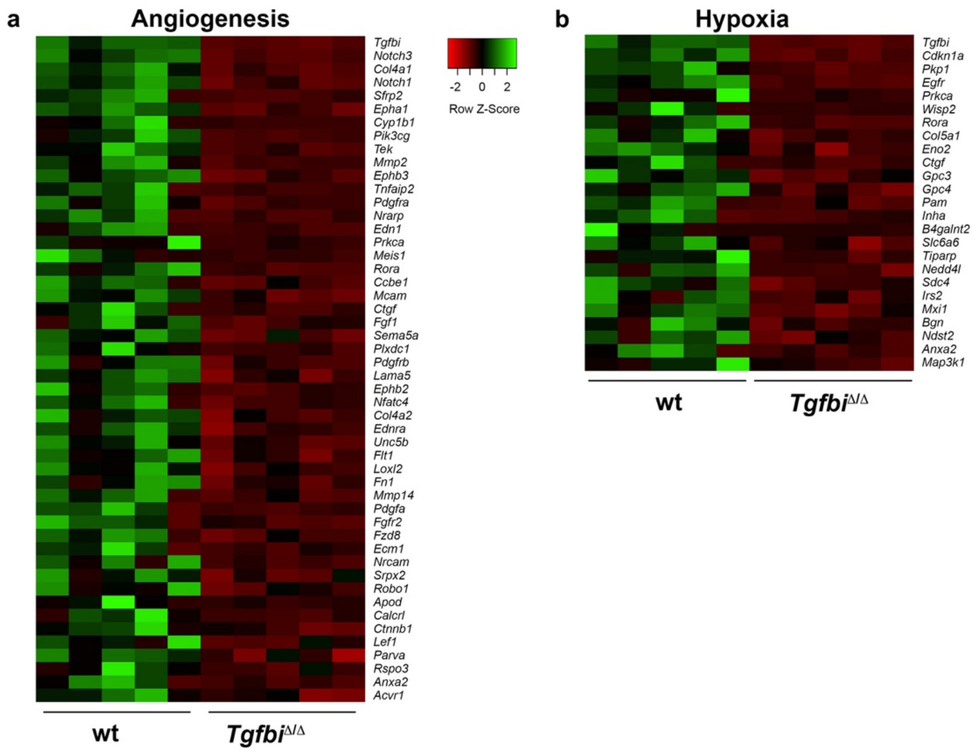

Supplemental File 3

Supplemental File 3: Heatmaps of statistically significant changed genes from the enrichment score analyses showed in Figure 2a.

a **Hypoxia**

id	genename	ENSMUSG	baseMean	log2FoldChange	lfcSE	pvalue	padj
9373	Tgfbi	ENSMUSG00000035493	549.354562	-2.3573141	0.18568498	6.28E-37	1.01E-32
3806	Cdkn1a	ENSMUSG00000023067	4096.48474	-1.2474963	0.16543155	4.67E-14	5.54E-12
5163	Pkp1	ENSMUSG00000026413	994.109497	-2.5224013	0.37005366	9.34E-12	8.40E-10
2206	Egfr	ENSMUSG00000020122	1574.60481	-0.9319081	0.17022852	4.39E-08	1.87E-06
659	Eno2	ENSMUSG00000004267	150.317145	-1.0246908	0.25483532	5.80E-05	0.00094569
14099	Gpc3	ENSMUSG00000055653	128.143564	-0.9234606	0.2546884	0.00028802	0.00358801
8240	Rora	ENSMUSG00000032238	596.122595	-0.7967052	0.19590147	4.76E-05	0.0008106
5408	Col5a1	ENSMUSG00000026837	2869.35252	-1.4367068	0.35456519	5.08E-05	0.00085113
7136	Slc6a6	ENSMUSG00000030096	2766.85829	-0.8356399	0.24721035	0.00072412	0.00759203
5127	Pam	ENSMUSG00000026335	2929.78539	-0.8345722	0.24035561	0.00051614	0.00576574
8584	Inha	ENSMUSG00000032968	32.763768	-2.5130386	0.72636108	0.00054065	0.00598112
7627	Gpc4	ENSMUSG00000031119	1340.30201	-0.5657171	0.16259615	0.00050277	0.00565588
2136	Ctgf	ENSMUSG00000019997	4562.66334	-1.1735269	0.31190321	0.00016824	0.00232576
10523	Irs2	ENSMUSG00000038894	547.67702	-1.2231683	0.38870186	0.00165066	0.01477289
17432	Srpx	ENSMUSG00000090084	82.5005644	-0.647171	0.2570284	0.01180579	0.06431101
7757	Bgn	ENSMUSG00000031375	6607.99812	-0.8064399	0.26387415	0.00224196	0.01874118
10681	Ndst2	ENSMUSG00000039308	619.449446	-0.5221891	0.18333598	0.00439585	0.03110912
16379	Kif5a	ENSMUSG00000074657	30.2243869	-0.71172	0.34521053	0.03923645	0.14894406
9092	Tiparp	ENSMUSG00000034640	500.278271	-0.8771992	0.26190585	0.00081018	0.00834515
2883	Gcnt2	ENSMUSG00000021360	346.05598	-0.7474049	0.2836372	0.00841191	0.05024546
5831	Wisp2	ENSMUSG00000027656	3421.52133	-3.2691958	0.79002581	3.50E-05	0.000625
4285	Nedd4l	ENSMUSG00000024589	2654.46045	-0.4605364	0.14126385	0.00111367	0.01081775
13356	Prkca	ENSMUSG00000050965	5394.53623	-1.8584092	0.43712444	2.12E-05	0.00040768
8237	Anxa2	ENSMUSG00000032231	15658.9651	-0.5526455	0.20410307	0.00677556	0.04289941
1681	Sdc4	ENSMUSG00000017009	13748.7755	-0.4454057	0.14072569	0.00155049	0.01411043
7140	Bhlhe40	ENSMUSG00000030103	3388.92074	-0.5746693	0.22434898	0.01042221	0.05851939
2637	Hoxb9	ENSMUSG00000020875	34.91473	-1.0496552	0.52124162	0.0440348	0.16031592
1457	Stc1	ENSMUSG00000014813	302.77821	-0.8206854	0.32985217	0.01284493	0.06835683
3087	Map3k1	ENSMUSG00000021754	2735.45526	-0.6731323	0.2535003	0.00792259	0.04827745
13776	Kdm3a	ENSMUSG00000053470	2287.61908	-0.4410421	0.19353072	0.02267148	0.10237427
9309	Tpbg	ENSMUSG00000035274	800.545744	-0.4743691	0.19505169	0.01501499	0.07672334
8144	Ets1	ENSMUSG00000032035	1602.64653	-0.738877	0.28123058	0.00860655	0.05108403
14353	Chst3	ENSMUSG00000057337	23.9801137	-1.0648151	0.48578188	0.02838229	0.119495
6029	Efna3	ENSMUSG00000028039	23.1918393	-0.7108226	0.40537365	0.07951624	0.23360176
14351	Bcl2	ENSMUSG00000057329	1029.35035	-0.6728887	0.26029652	0.00973549	0.05577873
4145	Dtna	ENSMUSG00000024302	262.436746	-0.4228546	0.21678682	0.05111002	0.17736164
722	Pgf	ENSMUSG00000004791	2386.66592	-0.706696	0.34995449	0.04344627	0.15900502
4783	Gaa	ENSMUSG00000025579	6442.31637	-0.6896884	0.28295114	0.01478993	0.07588374
8521	Ilvbl	ENSMUSG00000032763	829.807552	-0.4787939	0.21829308	0.0282819	0.11919765
1361	B4galnt2	ENSMUSG00000013418	64.2024938	-3.9407372	1.16482699	0.0007167	0.00754602
2105	Dcn	ENSMUSG00000019929	1739.00108	-0.6236195	0.28827698	0.0305208	0.12553366
6081	F3	ENSMUSG00000028128	3086.47558	-0.5345539	0.27283042	0.05007883	0.17473016
4512	Mxi1	ENSMUSG00000025025	1703.11822	-0.3403339	0.11057183	0.00208434	0.01770085
9468	Ids	ENSMUSG00000035847	1043.74098	-0.5346891	0.27089719	0.04840776	0.17091121
808	Hmox1	ENSMUSG00000005413	1105.62214	-0.4508029	0.2375241	0.05770599	0.19208931
9845	Prkcdbp	ENSMUSG00000037060	995.694043	-0.5693883	0.32465373	0.07945921	0.23351994
395	Ncan	ENSMUSG00000002341	2.8581936	-0.9832643	0.94423077	0.29771825	0.52286052
5055	Ankzf1	ENSMUSG00000026199	587.545213	-0.3929813	0.22435017	0.07983531	0.23423819
8953	Gpc1	ENSMUSG00000034220	812.157604	-0.5132825	0.29357771	0.08039994	0.23505251
2835	Tgfb3	ENSMUSG00000021253	6971.167	-0.3087597	0.15179491	0.04194573	0.15532436

b **Angiogenesis**

id	genename	ENSMUSG	baseMean	log2FoldChange	lfcSE	pvalue	padj
9373	Tgfbi	ENSMUSG00000035493	549.35456	-2.357314	0.185685	6.28E-37	1.01E-32
10218	Notch3	ENSMUSG00000038146	2203.2155	-1.767125	0.2055472	8.17E-18	1.92E-15
5451	Notch1	ENSMUSG00000026923	3725.2157	-1.22157	0.1970508	5.67E-10	3.68E-08
7825	Col4a1	ENSMUSG00000031502	11789.209	-1.322018	0.2108079	3.58E-10	2.42E-08
7053	Epha1	ENSMUSG00000029859	989.77209	-0.85996	0.1431346	1.88E-09	1.09E-07
889	Ephb3	ENSMUSG00000005958	1794.0019	-0.85247	0.1729243	8.23E-07	2.44E-05
951	Tek	ENSMUSG00000006386	106.36587	-1.590362	0.3131849	3.81E-07	1.26E-05
5999	Sfrp2	ENSMUSG00000027996	893.16975	-2.944951	0.4862587	1.39E-09	8.35E-08
2455	Pik3cg	ENSMUSG00000020573	585.02078	-1.240827	0.2412717	2.71E-07	9.37E-06
7892	Ednra	ENSMUSG00000031616	252.54017	-0.785771	0.2246928	0.0004704	0.0053426
7960	Mmp2	ENSMUSG00000031740	1967.2459	-1.463137	0.2963419	7.92E-07	2.37E-05
6716	Pdgfra	ENSMUSG00000029231	336.67634	-1.358387	0.3004101	6.13E-06	0.000139
8204	Mcam	ENSMUSG00000032135	1873.6319	-0.707603	0.1792823	7.92E-05	0.001233
3340	Sema5a	ENSMUSG00000022231	207.08881	-1.310903	0.3504426	0.0001835	0.0024894
12462	Ccbe1	ENSMUSG00000046318	77.950669	-1.870706	0.460817	4.92E-05	0.0008294
6949	Flt1	ENSMUSG00000029648	369.59418	-0.786885	0.2283581	0.0005693	0.0062356
8240	Rora	ENSMUSG00000032238	596.12259	-0.796705	0.1959015	4.76E-05	0.0008106
4301	Pdgfrb	ENSMUSG00000024620	635.53172	-0.995381	0.2675232	0.0001986	0.0026566
2468	Nrcam	ENSMUSG00000020598	19.006895	-1.477879	0.4696528	0.001651	0.0147729
2858	Tnfaip2	ENSMUSG00000021281	2782.4514	-2.393061	0.4899583	1.04E-06	2.96E-05
1549	Lama5	ENSMUSG00000015647	2710.0982	-0.668571	0.1808547	0.0002184	0.0028774
9783	Fzd8	ENSMUSG00000036904	160.88589	-0.803877	0.2488602	0.0012369	0.0117717
7826	Col4a2	ENSMUSG00000031503	5365.5852	-0.835434	0.2351008	0.0003801	0.0045041
16610	Nrarp	ENSMUSG00000078202	900.87448	-2.169548	0.5024638	1.58E-05	0.0003157
3875	Nfatc4	ENSMUSG00000023411	145.18885	-1.165456	0.3236698	0.0003173	0.0038684
2890	Edn1	ENSMUSG00000021367	230.59071	-1.667253	0.3872453	1.67E-05	0.0003298
6374	Ephb2	ENSMUSG00000028664	53.59702	-1.398816	0.3830061	0.00026	0.0032928
2193	Unc5b	ENSMUSG00000020099	773.74185	-0.723018	0.2080386	0.0005101	0.0057119
8945	Loxl2	ENSMUSG00000034205	559.43798	-0.980399	0.2854736	0.0005941	0.0064785
1713	Plxdc1	ENSMUSG00000017417	69.758101	-1.32815	0.3556645	0.0001883	0.0025474
9682	Fgf1	ENSMUSG00000036585	86.676493	-1.506335	0.4023279	0.0001811	0.0024649
5051	Fn1	ENSMUSG00000026193	4789.7017	-1.181776	0.3445403	0.0006036	0.0065683
150	Mmp14	ENSMUSG00000000957	4793.2027	-1.027548	0.3012345	0.000647	0.0069741
4872	Pdgfa	ENSMUSG00000025856	335.63615	-0.944878	0.2892126	0.0010867	0.0106069
2136	Ctgf	ENSMUSG00000019997	4562.6633	-1.173527	0.3119032	0.0001682	0.0023258
7528	Fgfr2	ENSMUSG00000030849	149.95597	-1.382603	0.4261975	0.0011785	0.0113098
4026	Cyp1b1	ENSMUSG00000024087	704.93821	-2.669326	0.502931	1.11E-07	4.29E-06
7696	Srpx2	ENSMUSG00000031253	190.66627	-0.874022	0.29783	0.0033394	0.0252635
2229	Meis1	ENSMUSG00000020160	151.55568	-1.98416	0.478091	3.32E-05	0.0005969
5407	Acvr1	ENSMUSG00000026836	705.69813	-0.511719	0.1917336	0.0076098	0.0468532
4673	Mmp19	ENSMUSG00000025355	170.02617	-0.606225	0.2492737	0.0150171	0.0767233
6071	Ecm1	ENSMUSG00000028108	969.41742	-1.294183	0.4014668	0.0012657	0.0120107
1025	Ctnnb1	ENSMUSG00000006932	21402.626	-0.529149	0.1892255	0.0051677	0.0349946
7484	Parva	ENSMUSG00000030770	4293.7347	-0.475518	0.1724348	0.0058215	0.0382582
14648	Calcrl	ENSMUSG00000059588	146.39951	-0.709918	0.2525247	0.0049345	0.0337858
13356	Prkca	ENSMUSG00000050965	5394.5362	-1.858409	0.4371244	2.12E-05	0.0004077
5995	Lef1	ENSMUSG00000027985	37.879304	-1.356607	0.4892963	0.0055616	0.0370033
3708	Robo1	ENSMUSG00000022883	252.74933	-1.092661	0.3792699	0.0039647	0.0287831
8237	Anxa2	ENSMUSG00000032231	15658.965	-0.552646	0.2041031	0.0067756	0.0428994
18133	Vezf1	ENSMUSG00000098546	1738.5972	-0.465776	0.1971258	0.0181358	0.0876562
7133	Wnt7a	ENSMUSG00000030093	6.0060425	-1.740204	0.8746468	0.0466341	0.1664108
5543	Slc12a6	ENSMUSG00000027130	513.40785	-0.559752	0.243921	0.0217442	0.099225
10659	Tgfb2	ENSMUSG00000039239	1420.3917	-0.625296	0.2512357	0.0128145	0.0682644
11372	Mmrn2	ENSMUSG00000041445	236.78561	-0.456181	0.2112416	0.0308098	0.1263327
8144	Ets1	ENSMUSG00000032035	1602.6465	-0.738877	0.2812306	0.0086065	0.051084
14627	Bcas3	ENSMUSG00000059439	585.46446	-0.402781	0.1635321	0.0137776	0.072005
83	Acvrl1	ENSMUSG00000000530	998.90699	-0.465672	0.2324265	0.045121	0.1631203
2087	Rspo3	ENSMUSG00000019880	69.195072	-1.196095	0.4396949	0.0065226	0.0417153
8026	Cdh13	ENSMUSG00000031841	192.6493	-0.722698	0.3060675	0.0182141	0.0878376
12173	Setd2	ENSMUSG00000044791	1871.9907	-0.641592	0.2747387	0.0195287	0.0921895
6433	Hspg2	ENSMUSG00000028763	1733.7802	-0.593245	0.3208227	0.064438	0.2060162
722	Pgf	ENSMUSG00000004791	2386.6659	-0.706696	0.3499545	0.0434463	0.159005
7389	Mfge8	ENSMUSG00000030605	54587.208	-1.268443	0.5423657	0.0193498	0.0915614
7862	Slit2	ENSMUSG00000031558	88.633984	-0.856737	0.3710687	0.0209527	0.0969071
7981	Cx3cl1	ENSMUSG00000031778	3891.3481	-0.500255	0.222675	0.0246675	0.1083935
2333	Flt4	ENSMUSG00000020357	173.13655	-0.553109	0.2534084	0.0290594	0.1212044
9917	Ovol2	ENSMUSG00000037279	144.40912	-0.732298	0.3244961	0.0240255	0.1063899
14592	Lep	ENSMUSG00000059201	0.9522537	-2.703631	2.1093185	0.1999283	NA
3521	Apod	ENSMUSG00000022548	1963.1835	-2.932471	1.0194014	0.0040191	0.0290463
8475	Thsd7a	ENSMUSG00000032625	12.260188	-0.698045	0.5150048	0.1752853	0.3827009
4861	Nrp1	ENSMUSG00000025810	568.27977	-0.387895	0.2163402	0.0729748	0.2220453
7866	Fgfr1	ENSMUSG00000031565	5256.3197	-0.692173	0.3122771	0.0266548	0.1143329
1203	Vav2	ENSMUSG00000009621	770.12676	-0.597977	0.2898014	0.0390745	0.1485405
808	Hmox1	ENSMUSG00000005413	1105.6221	-0.450803	0.2375241	0.057706	0.1920893
666	Hif3a	ENSMUSG00000004328	2.4068205	-1.705147	1.2848613	0.1844738	0.3954536
234	Col18a1	ENSMUSG00000001435	1814.1474	-0.383453	0.2291556	0.094262	0.2599839
1276	Plcd1	ENSMUSG00000010660	430.34995	-0.398879	0.2244033	0.0754843	0.2261984
5729	Angpt4	ENSMUSG00000027460	17.760951	-0.655944	0.47479	0.1671111	0.3713889
89	Itga5	ENSMUSG00000000555	521.91167	-0.402673	0.2432014	0.0977793	0.2659187
8131	Thy1	ENSMUSG00000032011	586.27915	-0.367139	0.2233932	0.1002868	0.2696324
7838	Vegfc	ENSMUSG00000031520	159.28676	-0.854518	0.5116393	0.0948885	0.2609922
5531	Itgav	ENSMUSG00000027087	2704.635	-0.785083	0.3509642	0.0252909	0.1105259
11652	Esm1	ENSMUSG00000042379	495.08272	-1.402768	0.5374062	0.0090474	0.0530843
2838	Vash1	ENSMUSG00000021256	430.09801	-0.345754	0.2202036	0.1163792	0.29731
6031	Adam15	ENSMUSG00000028041	1869.7668	-0.301594	0.193463	0.1190141	0.3018343
8570	Cspg4	ENSMUSG00000032911	140.61656	-0.543496	0.3249271	0.094392	0.2602528
7119	Prok2	ENSMUSG00000030069	0.8319637	-2.283911	2.1442105	0.286807	NA
12406	Pofut1	ENSMUSG00000046020	910.44827	-0.290585	0.1823122	0.1109619	0.2883367
14630	Ptk2b	ENSMUSG00000059456	685.67686	-0.344917	0.238201	0.1476146	0.3444313
8649	Tie1	ENSMUSG00000033191	314.33066	-0.300215	0.2223916	0.1770353	0.3851556
3044	Aggf1	ENSMUSG00000021681	1724.9758	-0.282284	0.17197	0.100699	0.2703185
14349	Arhgap24	ENSMUSG00000057315	150.21798	-0.318365	0.2298389	0.166001	0.3700522

c **EMT**

id	genename	ENSMUSG	baseMean	log2FoldChange	lfcSE	pvalue	padj
9373	Tgfbi	ENSMUSG00000035493	549.35456	-2.3573141	0.185685	6.28E-37	1.01E-32
7825	Col4a1	ENSMUSG00000031502	11789.209	-1.3220182	0.2108079	3.58E-10	2.42E-08
2270	Col6a2	ENSMUSG00000020241	3295.232	-1.5458862	0.29548	1.68E-07	6.22E-06
659	Eno2	ENSMUSG00000004267	150.31715	-1.0246908	0.2548353	5.80E-05	0.0009457
5915	Ptx3	ENSMUSG00000027832	369.33686	-1.9922911	0.4310166	3.79E-06	9.26E-05
13585	Lrrc15	ENSMUSG00000052316	1168.1699	-2.6373474	0.4037509	6.48E-11	5.01E-09
1508	Gadd45b	ENSMUSG00000015312	975.42243	-1.5851945	0.3306743	1.64E-06	4.41E-05
7960	Mmp2	ENSMUSG00000031740	1967.2459	-1.4631367	0.2963419	7.92E-07	2.37E-05
1802	Pmp22	ENSMUSG00000018217	645.43111	-1.093752	0.2655653	3.81E-05	0.0006684
248	Col1a1	ENSMUSG00000001506	23458.393	-1.7498753	0.3868546	6.09E-06	0.0001386
5937	Notch2	ENSMUSG00000027878	910.31422	-0.8816746	0.2381741	0.0002141	0.0028274
5874	Postn	ENSMUSG00000027750	8134.0654	-1.7464487	0.3526174	7.31E-07	2.21E-05
13940	Adam12	ENSMUSG00000054555	311.31466	-1.7257262	0.3532869	1.04E-06	2.96E-05
2319	Fstl3	ENSMUSG00000020325	513.49826	-0.9462939	0.2520051	0.0001733	0.0023731
8360	Nt5e	ENSMUSG00000032420	787.99645	-0.7227693	0.1807794	6.39E-05	0.0010264
6959	Col1a2	ENSMUSG00000029661	26100.717	-1.5653027	0.3441768	5.42E-06	0.0001255
4301	Pdgfrb	ENSMUSG00000024620	635.53172	-0.9953814	0.2675232	0.0001986	0.0026566
9783	Fzd8	ENSMUSG00000036904	160.88589	-0.8038771	0.2488602	0.0012369	0.0117717
7826	Col4a2	ENSMUSG00000031503	5365.5852	-0.8354337	0.2351008	0.0003801	0.0045041
5408	Col5a1	ENSMUSG00000026837	2869.3525	-1.4367068	0.3545652	5.08E-05	0.0008511
4972	Col5a2	ENSMUSG00000026042	4003.5319	-1.0569263	0.2823925	0.000182	0.0024734
917	Htra1	ENSMUSG00000006205	417.99073	-1.8567674	0.4571405	4.87E-05	0.0008244
12826	Col6a3	ENSMUSG00000048126	1566.0064	-1.0858456	0.3188057	0.0006593	0.0070687
5901	Pfn2	ENSMUSG00000027805	1036.8441	-1.897375	0.4279607	9.27E-06	0.0001989
15238	Fbln2	ENSMUSG00000064080	3395.934	-1.2919454	0.3454982	0.0001845	0.0025006
8945	Loxl2	ENSMUSG00000034205	559.43798	-0.9803989	0.2854736	0.0005941	0.0064785
14222	Slit3	ENSMUSG00000056427	238.83943	-1.4470497	0.3902402	0.0002088	0.0027701
7926	Cdh11	ENSMUSG00000031673	692.38061	-1.2021882	0.3008166	6.43E-05	0.0010325
8298	Col12a1	ENSMUSG00000032332	5565.0013	-1.2800507	0.3539931	0.0002992	0.0036979
5051	Fn1	ENSMUSG00000026193	4789.7017	-1.1817756	0.3445403	0.0006036	0.0065683
4291	Fbn2	ENSMUSG00000024598	2246.064	-4.7647795	0.8743926	5.06E-08	2.14E-06
150	Mmp14	ENSMUSG00000000957	4793.2027	-1.0275476	0.3012345	0.000647	0.0069741
10986	Lrp1	ENSMUSG00000040249	1286.6233	-0.7613668	0.2754401	0.0057066	0.0376703
2136	Ctgf	ENSMUSG00000019997	4562.6633	-1.1735269	0.3119032	0.0001682	0.0023258
11927	Mmp3	ENSMUSG00000043613	478.80337	-1.5245577	0.5273324	0.0038393	0.0281019
1869	Sparc	ENSMUSG00000018593	20084.865	-0.668741	0.2330808	0.0041159	0.0295361
10328	Pmepa1	ENSMUSG00000038400	18166.468	-0.8476592	0.2747633	0.0020352	0.0173851
7757	Bgn	ENSMUSG00000031375	6607.9981	-0.8064399	0.2638742	0.002242	0.0187412
2465	Sdc1	ENSMUSG00000020592	4267.2154	-0.5153053	0.1298604	7.24E-05	0.0011446
3262	Bmp1	ENSMUSG00000022098	1731.172	-0.5981615	0.2266262	0.0083048	0.0498843
644	Col5a3	ENSMUSG00000004098	558.48427	-1.172563	0.3402345	0.0005682	0.0062304
3913	Thbs2	ENSMUSG00000023885	3325.8049	-1.4271109	0.379932	0.0001725	0.002369
9303	Abi3bp	ENSMUSG00000035258	283.36051	-1.3864305	0.4116	0.0007561	0.0078849
6071	Ecm1	ENSMUSG00000028108	969.41742	-1.2941829	0.4014668	0.0012657	0.0120107
5258	Prrx1	ENSMUSG00000026586	296.66916	-0.7962728	0.2793027	0.0043591	0.0308902
2002	Slc6a8	ENSMUSG00000019558	2198.5466	-0.5357632	0.1759826	0.0023314	0.0193437
9639	Lum	ENSMUSG00000036446	880.18585	-1.2876624	0.3777981	0.0006536	0.007041
4973	Col3a1	ENSMUSG00000026043	17554.538	-1.1840539	0.3500687	0.0007187	0.007559
2096	Lama2	ENSMUSG00000019899	65.012649	-0.8723059	0.3395263	0.0101938	0.0576616
948	Fbln1	ENSMUSG00000006369	346.79759	-0.882743	0.3328486	0.0079996	0.048585
4203	Lama3	ENSMUSG00000024421	167.89245	-1.2825888	0.4242024	0.0024984	0.020302
1681	Sdc4	ENSMUSG00000017009	13748.775	-0.4454057	0.1407257	0.0015505	0.0141104
4146	Cdh2	ENSMUSG00000024304	81.593876	-0.834318	0.3531581	0.0181546	0.0876944
13691	Gas1	ENSMUSG00000052957	591.4583	-0.8191954	0.2993466	0.0062075	0.0402445
9049	Edil3	ENSMUSG00000034488	160.24057	-1.1769547	0.3992615	0.0032001	0.0244407
13352	Gja1	ENSMUSG00000050953	4778.6777	-0.8851801	0.3303464	0.0073721	0.0457235
10377	Rgs4	ENSMUSG00000038530	104.5044	-0.7966981	0.3587267	0.0263574	0.1135355
7731	Flna	ENSMUSG00000031328	10931.351	-0.4831179	0.2265786	0.0329879	0.1328143
6200	Tnc	ENSMUSG00000028364	4667.427	-1.2011999	0.450485	0.0076655	0.0471249
15573	Myl9	ENSMUSG00000067818	376.07909	-0.562319	0.2765925	0.0420501	0.1555671
7862	Slit2	ENSMUSG00000031558	88.633984	-0.856737	0.3710687	0.0209527	0.0969071
15841	Serpinh1	ENSMUSG00000070436	13354.558	-0.4963781	0.2306643	0.0314012	0.1280691
4442	Efemp2	ENSMUSG00000024909	1357.0157	-0.436607	0.2165209	0.0437513	0.1596106
5578	Fbn1	ENSMUSG00000027204	1250.5508	-0.7573532	0.3434789	0.0274577	0.1166596
5987	Col11a1	ENSMUSG00000027966	13749.247	-1.2092562	0.4580644	0.0082924	0.0498843
2105	Dcn	ENSMUSG00000019929	1739.0011	-0.6236195	0.288277	0.0305208	0.1255337
6992	Pcolce	ENSMUSG00000029718	735.23712	-0.6117624	0.2972806	0.0396034	0.1498035
3115	Nid2	ENSMUSG00000021806	409.1294	-0.6035915	0.2944109	0.0403481	0.1516535
4238	Dpysl3	ENSMUSG00000024501	327.55151	-0.385363	0.2334769	0.0988325	0.2676077
11339	Inhba	ENSMUSG00000041324	62.311311	-0.7551554	0.4058482	0.0627888	0.2024961
11931	Ecm2	ENSMUSG00000043631	51.808662	-0.6445425	0.3607561	0.0739952	0.2236211
6968	Eln	ENSMUSG00000029675	2113.5314	-1.1548465	0.5102985	0.0236307	0.1050197

Supplemental File 4

Supplemental File 4: List of genes found to have a positive enrichment score in the analyses of hallmark hypoxia, GO angiogenesis and hallmark EMT in the RNAseq of tumours coming from MMTV-PyMT;*Tgfbi*$^{+/+}$ and MMTV-PyMT;*Tgfbi*$^{\Delta/\Delta}$ mice.

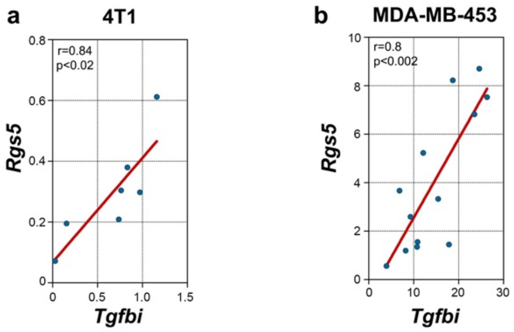

Supplemental File 5

Supplemental File 5: Correlation analyses of *Rgs5* and *Tgfbi* transcripts in 4T1 (n=7) and MDA-MB-453 (n=13) tumours. Tumours were pulverised, their RNA was extracted, subjected to reverse transcription, and analysed by qPCR for the expression of *Rgs5* and *Tgfbi*. *Rplp0* was used as a housekeeping gene for mouse and *SNRPD3* for human.

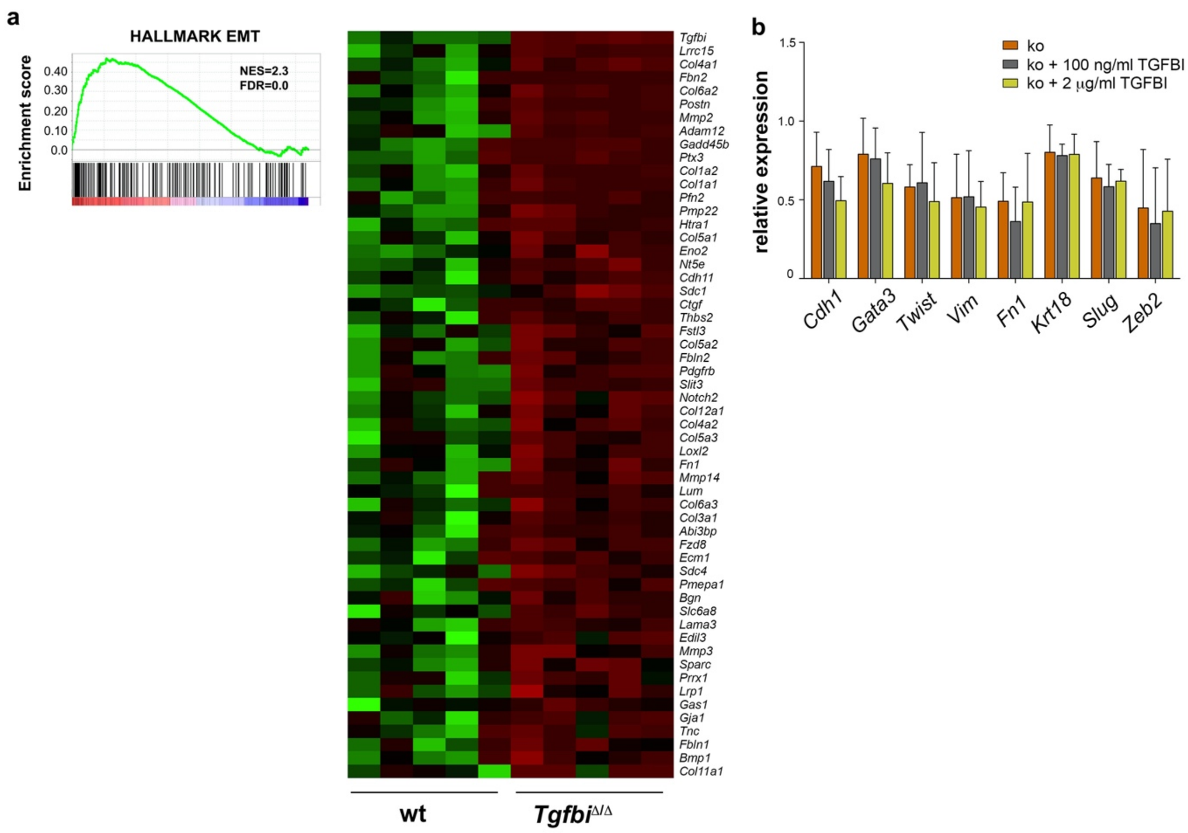

Supplemental File 6

Supplemental File 6: (a) Enrichment score analysis of MMTV-PyMT;*Tgfbi*$^{+/+}$ and MMTV-PyMT;*Tgfbi*$^{\Delta/\Delta}$ tumours for Hallmark EMT (n=5) and heatmap of statistically significant changed genes. (b) qPCR of MMTV-PyMT;*Tgfbi*$^{\Delta/\Delta}$ tumour cells grown with either 100 ng/ml or 2 μg/ml exogenous TGFBI. Data were analysed using one-way ANOVA, and are presented as mean and SD (n=5). *Rplp0* was used as a housekeeping gene.

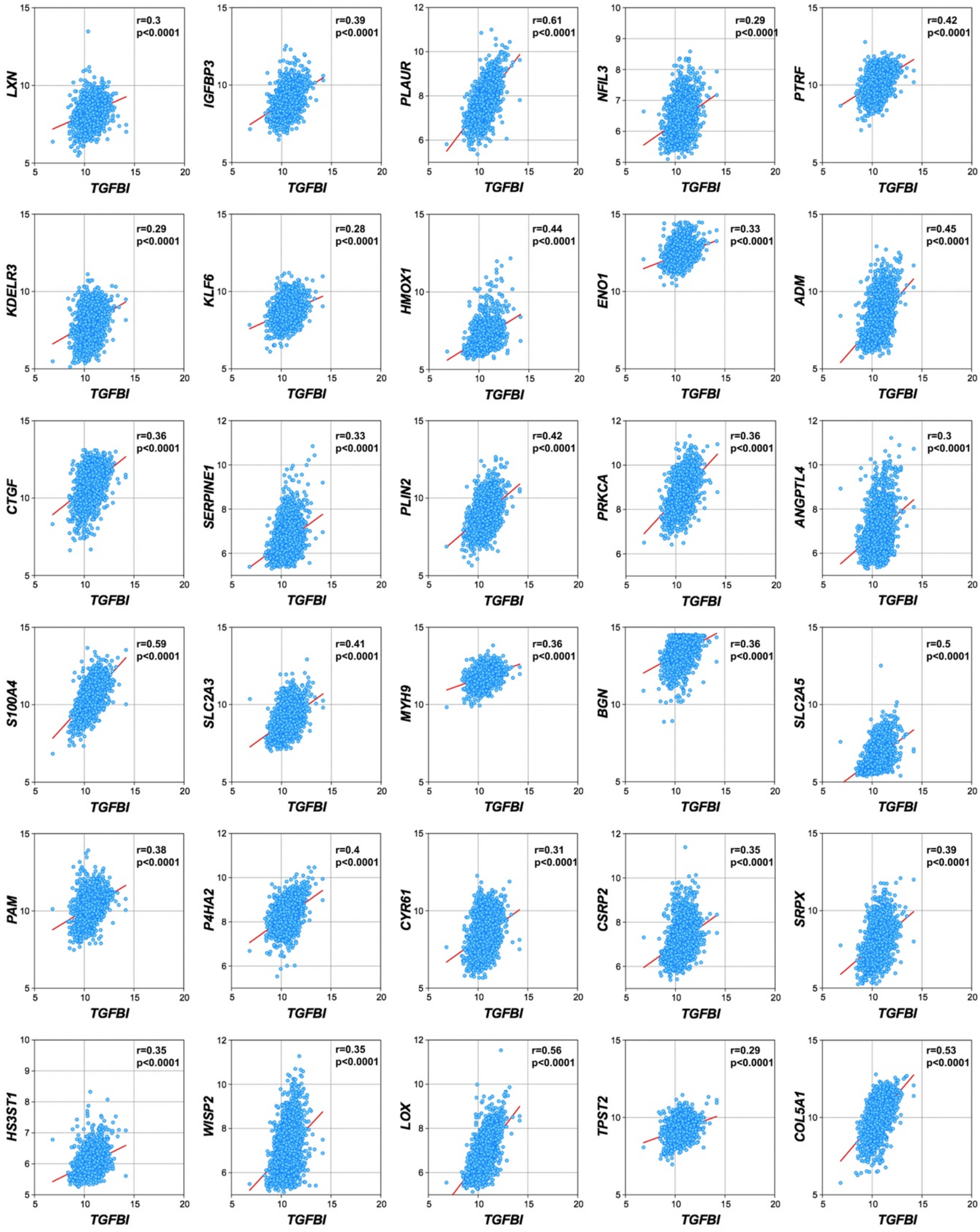

Supplemental File 7

Supplemental File 7: Spearman's correlation of hypoxia-related genes with *TGFBI* in the METABRIC cohort.

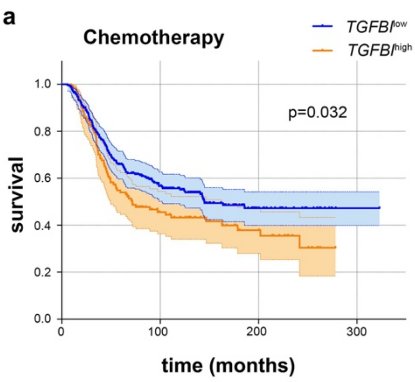

Supplemental File 8

Supplemental File 8: Survival curves of breast cancer patients in the METABRIC cohort who received chemotherapy classified according to the expression of *TGFBI*. Patients were stratified using the application *Evaluate cutpoints*, and the survival curves were compared using the Log-rank (Mantel-Cox) test (n_{high}=123, n_{low}=250).

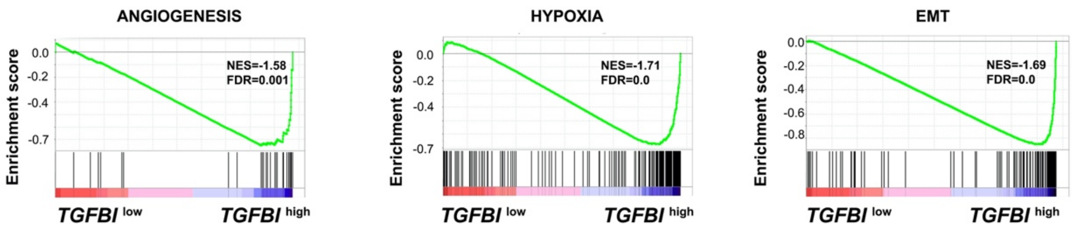

Supplemental File 9

Supplemental File 9: Enrichment score analyses of *TGFBI*^{low} (<75th percentile) and *TGFBI*^{high} (≥75th percentile) tumours in the METABRIC

Supplemental File 10. Table 1: Primer Sets Used in This Study.

ID	Forward 5'-3'	Reverse 5'-3'
Rplp0	GATTCGGGATATGCTGTTGG	GTTCTGAGCTGGCACAGTGA
Krt18	CGAGGCACTCAAGGAAGAAC	AATCTGGGCTTCCAGACCTT
Aldh1a3	GAGCGATCCTGGCTACTCTG	GACGAAAAAGGCATGAAGGA
Tgfbi	ATCACCAACAACATCCAGCA	CCAGCACGGTATTGAGTCCT
TGFBI	AAGTCTCTCCAAGGTGACAAGC	CCTTGTTGACACTCACCACATT
SNRPD3	GCCCAGATTGTGAAGGACAT	TCAGCTCAGCACGGTAGTTG
Gata3	GCTACGGTGCAGAGGTATCC	AGAGATCCGTGCAGCAGAG
Mmp2	ACACTGGGACCTGTCACTCC	TGTCACTGTCCGCCAAATAA
Mmp9	CCAGATGATGGGAGAGAAGC	TTGAGGCCTTTGAAGGTTTG
Snai2	GAACCCACACATTGCCTTGT	GCAGAAGCGACATTCTGGAG
Zeb2	CTATTCCCCTGCATCAGCAT	GGCTTGTCAGTCCTTTCTCG
Vim	GCGAGGAGAGCAGGATTTCTC	GGGTGTCAACCAGAGGAAGT
Fn1	TGCACGTGTGTGGGGAACGG	CCCGGCCCTGACCAAAGCAG
Rgs5	AGACGGTTCCACCAGGTTC	CAGACAGAGGCCCTAAAGA
Hif1a	CAGTCGACACAGCCTCGATA	CGGCTCATAACCCATCAACT
